# Safety of multiple intravenous infusions of adipose-derived mesenchymal stem cells for hospitalized cases of COVID-19: a randomized controlled trial

**DOI:** 10.3389/fmed.2023.1321303

**Published:** 2023-12-22

**Authors:** Constanza de Dios, Ridhima Vij, Hosu Kim, Hyeonggeun Park, Donna Chang

**Affiliations:** ^1^Department of Psychiatry and Behavioral Sciences, University of Texas Health Science Center, Houston, TX, United States; ^2^Hope Biosciences Research Foundation, Sugar Land, TX, United States; ^3^Hope Biosciences, Sugar Land, TX, United States

**Keywords:** mesenchymal stem cells, adipose-derived, intravenous, safety, COVID-19

## Abstract

**Objective:**

The purpose of the study was to assess the safety of allogeneic, Hope Biosciences Adipose Derived Mesenchymal Stem Cells (HB-adMSCs) for the treatment of hospitalized subjects with COVID-19.

**Methods:**

*N* = 48 patients were randomly assigned to HB-adMSC (100 MM) or placebo group. Four intravenous infusions of HB-adMSCs or saline were administered at days 0, 3, 7, 10. The primary safety endpoint was incidence of adverse and serious adverse events (AE/SAEs); secondary endpoints were incidence of specific AEs and alterations in hematology, biochemistry, and coagulation parameters.

**Results:**

Majority of AEs were mild in severity. HB-adMSC group showed a higher incidence of cardiopulmonary failure, anemia, anxiety, and diarrhea, while placebo group showed a higher incidence of headaches, fatigue, and chest discomfort (posterior probabilities ≥80%). Deaths were attributed to severe complications due to COVID-19 and were unrelated to study drug. No AEs were attributed to the treatment. Hematology and coagulation panel alterations were not associated with HB-adMSCs. Analyses of inflammatory markers showed increased levels of interleukin-6 and C-reactive protein over time in HB-adMSC group (posterior probabilities ≥78%).

**Conclusion:**

Multiple infusions of 100MM allogeneic HB-adMSCs were considered safe for the study population. More research is needed to determine the safety of MSC therapy.

**Clinical trial registration:**

(www.ClinicalTrials.gov) identifier NCT04362189.

## 1 Introduction

Coronavirus disease 2019 (COVID-19), known to be caused by Severe Acute Respiratory Syndrome Corona Virus-2 (SARS-CoV-2) was first identified in Wuhan, China in December 2019, and was soon recognized as a global pandemic (1). Following SARS-CoV-2 infection, the viral spike glycoprotein interacts with the host angiotensin-converting enzyme 2 (ACE2) receptor, typically expressed on alveolar epithelial cells forming SARS-CoV spike γlycoprotein-ACE2 complex (2). This cascade of events, coupled with SARS-CoV-2-induced damage to alveolar cells, triggers a systemic immune response releasing pro-inflammatory cytokines, including IL-1α, IL-6, TNF-α, and IFN-γ. This immune activation significantly contributes to the development of cytokine storm and plays a pivotal role in the onset of acute lung injury (3). The most common clinical manifestations of COVID-19 include mild or moderate respiratory symptoms that can lead to more severe complications such as acute respiratory distress syndrome (ARDS), pneumonia, cardiac arrhythmia, multiple organ damage and even death (4, 5). Pulmonary fibrosis risk is greatly increased in infected patients and may persist even after infection is resolved. Not just confined to the respiratory tract, there is evidence suggesting that coronaviruses have the potential to impact the central nervous system, causing manifestations such as headache, nausea, malaise as well as other neurological disease (6, 7).

Owing to its immunomodulatory and anti-inflammatory properties, mesenchymal stem cell (MSC) therapy has been previously proposed to be beneficial in the treatment of COVID-19 (8). Characterized by low immunogenicity, MSCs release key cytokines to intricately modulate immune responses. They effectively suppress inflammation and reduce cytokine storm, thereby promote regulatory cell expansion. In context of infection, MSCs play a crucial role in mitigating lung injury caused by inflammatory cascade. Significantly, MSCs have demonstrated efficacy in managing both acute and chronic inflammatory lung conditions by suppressing immune responses and potentially differentiating into type II alveolar epithelial cells during the reparative process (9–11).

Safety of MSCs for the treatment of COVID-19 has been consistently demonstrated across multiple clinical trials, utilizing MSCs from various sources (12–15). Notably, all previous studies reported no serious adverse events related to the therapy (8). Nevertheless, research is still ongoing to comprehensively understand their safety profiling. Here, we present safety of administration of multiple infusions of allogeneic Hope Biosciences adipose-derived MSCs (HB-adMSCs) to 48 hospitalized subjects with COVID-19 symptoms, randomized into treatment and placebo groups.

## 2 Materials and methods

The current trial was a phase II, placebo-controlled, double-blinded, randomized clinical trial to primarily determine the safety of multiple infusions of adipose-derived mesenchymal stem cells in hospitalized COVID-19 patients. The trial was conducted at two sites in Houston, Texas, from June 2020 to September 2021. Patients who met the following inclusion and exclusion criteria were enrolled into the study: *inclusion criteria* were (1) men and women, over 18 years of age, (2) suspected to have COVID-19 infection, (3) provided consent for participation, (4) agreed to collection of venous blood; *exclusion criteria* were (1) pregnancy, lactation and those who are not pregnant but do not take effective contraceptive measures, women of childbearing age, (2) patients who have participated or are participating in a clinical trial of an experimental vaccine for SARS-CoV-2 or coronavirus during the study or within 30 days, (3) inability to provide informed consent or to comply with test requirements (4) any medical disease or condition that, in the opinion of the site principal investigator or sub-investigator, precludes study participation, including acute, subacute, intermittent or chronic medical disease or condition that would place the subject at an unacceptable risk of injury, render the subject unable to meet the requirements of the protocol, or may interfere with the evaluation of responses or the subject’s successful completion of this trial (See Supplementary material for CONSORT checklist).

Four infusions each of either allogeneic HB-adMSCs or saline were administered intravenously (1-h long) at the rate of 83 gtts/min at days 0, 3, 7, and 10 to each of the eligible 48 subjects following screening, randomly allocated in a 3:2 ratio to each of the two groups: HB-adMSC (1×10^8^ cells) or placebo. Vital signs were recorded at minute 0, 15, 30, and 45 during the administration of each infusion, and then at minute 0, 30 and 60, post-infusion. Also, monitoring of any potential adverse event was performed by observing the subjects for at least 1 h after administration of infusion, for symptoms of infusion-related reactions and allergic reactions, including anaphylaxis. Subjects were also instructed for recognition of delayed serious allergic reactions and seeking for medical assistance if needed.

For HB-adMSCs production, emulsified fat from four qualified donors’ abdomen were extracted via liposuction procedure performed by a licensed physician. 4–7 mL of adipose tissues was then treated with collagenase to separate the stromal vascular fraction (SVF). The SVF was plated in Hope Biosciences’ (HB)-103 medium and the resulting adherent cells were further expanded in HB-101 medium to establish a P0 culture. Cells were cryopreserved at passages 0, 1 and 2 to create a complete bank of each donor. For infusions, passage 2 cells were thawed and cultured to passage 4. For each patient administration, 1.0 × 10^8^ HB-adMSCs were freshly harvested from passage 4 cultures and packaged in 10 mL 0.9% sterile saline. All infusions for each subject were produced using the same donor cells and administered within 96 h of packaging. To ensure safety and efficacy of the investigational drug, final product release criteria were set as follows; Viability (≥ 70%), Appearance (opaque white to faint yellow with no settlement), USP71 sterility (no organism seen), Mycoplasma (negative), Endotoxin (≤ 10 EU/mL), Gram stain (no organism seen) and Identity/purity by MSC-defining surface markers CD73 & CD29 (> 75%) and CD31 & CD45 (< 5%). All products provided for this study successfully met the cGMP compliant quality control standards. See Supplementary Table S6 for quality control metrics.

Following screening, each participant’s information was entered into a randomization spreadsheet (see Supplementary Table S7 for a sample randomization spreadsheet), with each subject categorized by risk level, pre-existing conditions, ethnicity, and age group. Randomization was performed by neutral personnel, and stratification categories were defined based on the following criteria: (1) illness severity – mild/severe, (2) pre-existing condition – yes/no, (3) ethnicity - African American or Hispanic/all other, and (4) age group - 18-41 years/older than 41 years. Treatment groups were identified as A and B. Group assignment was determined by the randomization spreadsheet, based on the four stratification variables listed above.

Amber bag covers were applied to saline infusion bags for product infusion. Only subject ID, date of birth (DOB), and expiration date were on the bag label, to ensure proper distribution. Mixer injected product into the bag, covered and applied label before handing off to the clinical team. Mixer also maintained product mixing log, which was only accessible to Mixer. Product was infused into an amber bag of 250 mL saline by Mixer. Pre-mixed bags were handed off to the clinical team who were blinded. The only individual with knowledge of bag contents was the designated mixer.

Primary safety endpoints included incidence of adverse (AE) and serious adverse events (SAEs). Secondary safety endpoints were incidence of specific AEs including serious infections, infusion-related reactions, hepatotoxicity, heart failure, and cytopenia. Safety was further assessed by evaluating alterations over time in laboratory parameters, including hematology (white blood cells, WBC; lymphocytes, platelets, neutrophils), biochemistry (comprehensive metabolic panel), and coagulation cascade (Prothrombin Time, PT; Partial Thromboplastin Time, PTT; and International Normalized Ratio, INR).

All randomized subjects who received at least one dose of HB-adMSCs infusion or placebo comprised the safety analysis set. Changes in coagulation parameters (WBC, lymphocytes, platelets, neutrophils %, prothrombin INR, PT, PTT) and inflammatory markers (Tumor Necrosis-alpha, TNF-α; interleukin-10; interleukin-6; and C-Reactive Protein, CRP) through time were modeled using generalized linear mixed models. Each model predicated each measure as a function of the interaction between the fixed factors treatment group and time (Infusions 1, 2, 3, 4), controlling for lower order effects of time and treatment group. Specific AEs/SAEs were analyzed in a generalized linear model, predicting the presence of a given AE/SAE as a function of treatment group.

Bayesian inference was used for analyses of incidence of specific AEs/SAEs and inflammatory markers; a posterior probability (PP) ≥75% constituted evidence for statistically reliable differences (see Supplementary material for Bayesian methods). Safety data for laboratory parameters were analyzed using frequentist inference. All analyses controlled for stratification variables (illness severity, pre-existing condition, ethnicity, age group). The study was approved by the Western IRB. All participants provided written informed consent.

The current trial was designed to accrue the largest possible sample size given available resources and anticipated demand for treatment and was approved to enroll up to *N* = 100 patients (HB-adMSC *N* = 60, placebo *N* = 40).

## 3 Results

### 3.1 Patient characteristics

**Figure 1:**
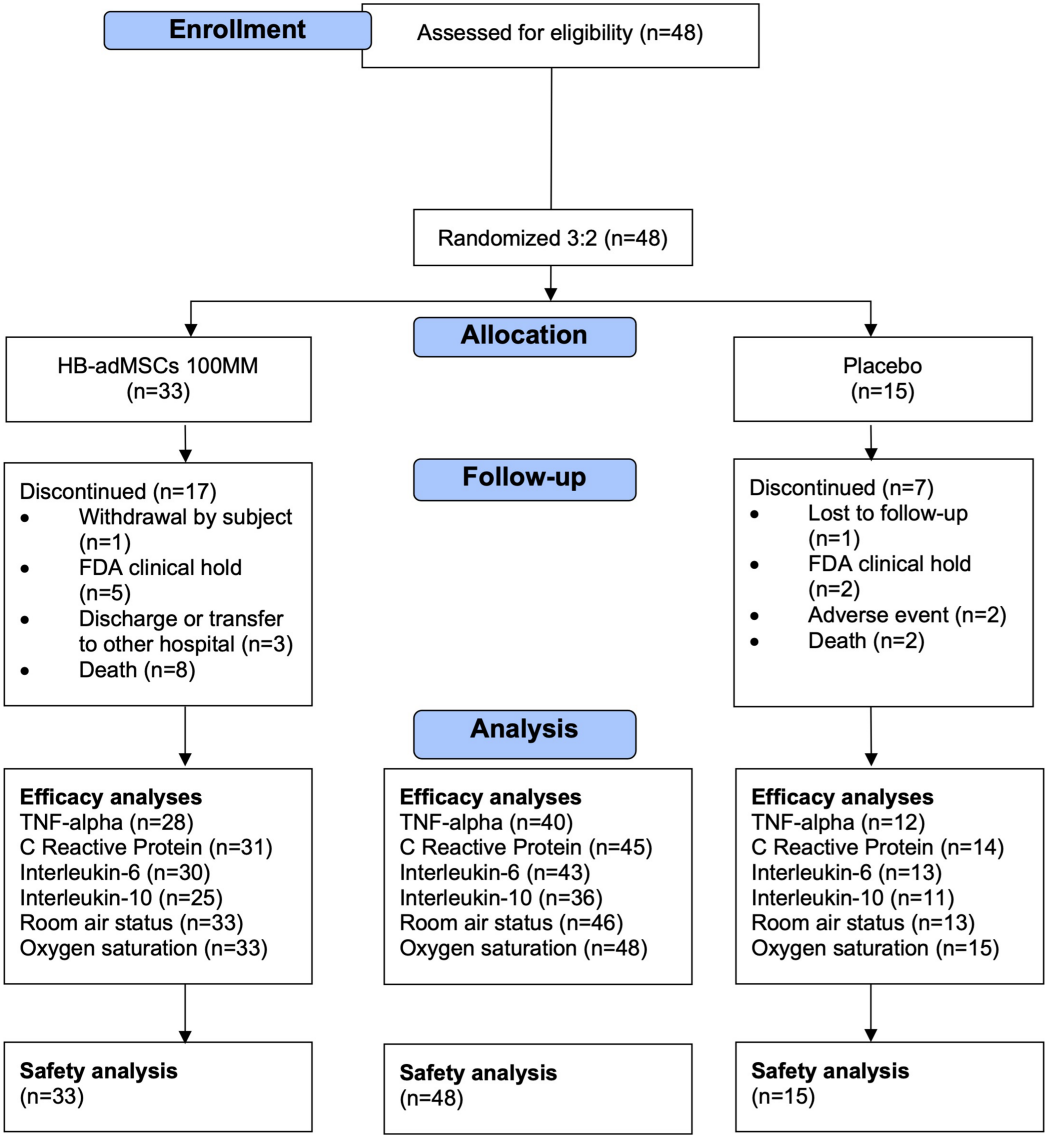
CONSORT flow diagram.

Forty-eight eligible patients were enrolled in the study and randomized into each group: HB-adMSC (*N* = 33) or placebo (*N* = 15) (Figure 1 shows the CONSORT flow diagram). Subjects were predominantly male (58.3%), with a mean age of 53.7 years, and consisted mostly of white (66.7%) individuals; 45.8% were of Hispanic/Latino ethnicity (Table 1). All subjects had at least one comorbidity, with an average of 3 comorbidities per subject. The most common comorbidities included hypertension and type II diabetes (Table 2). 95.8% of all subjects had at least one concomitant medication, with an average of 22.4 medications per subject (Table 2). HB-adMSC had greater incidence of comorbidities (incidence rate ratio 1.72, SE = 1.23, *p* = 0.008) and concomitant medications (incidence rate ratio 1.44, SE = 1.07, *p* < 0.001) than placebo at baseline. (See Supplementary Table S1 for complete list of comorbidities and medications).

**Table 1:** Demographic characteristics, comorbidities, concomitant medications, and adverse events by group.

Characteristic	HB-adMSCs 100MM, *N* = 33	Placebo, *N* = 15	Overall, *N* = 48
Age (years), M (SD)	53.2 (17.6)	54.7 (15.8)	53.7 (16.9)
Sex, n (%)			
Female	15 (45.5%)	5 (33.3%)	20 (41.7%)
Male	18 (54.5%)	10 (66.7%)	28 (58.3%)
Race, n (%)			
Asian	1 (3.0%)	1 (6.7%)	2 (4.2%)
Black or African American	11 (33.3%)	3 (20.0%)	14 (29.2%)
White	21 (63.6%)	11 (73.3%)	32 (66.7%)
Ethnicity, n (%)			
Hispanic or Latino	13 (39.4%)	9 (60.0%)	22 (45.8%)
Not Hispanic or Latino	20 (60.6%)	6 (40.0%)	26 (54.2%)
Weight (lbs), M (SD)	194.6 (48.2)	194.1 (43.7)	194.4 (46.3)
Height (in), M (SD)	66.5 (3.5)	66.1 (4.4)	66.4 (3.7)
BMI, M (SD)	30.8 (6.3)	31.2 (5.9)	30.9 (6.1)

HB-adMSCs 100MM, allogeneic Hope Biosciences adipose-derived MSCs 1×10^8^ cells.

**Table 2:** Comorbidities and concomitant medications by group.

Characteristic	HB-adMSCs 100MM, *N* = 33	Placebo, *N* = 15	Overall, *N* = 48
Comorbidities			
Number of comorbidities per subject, M (SD)	3.5 (2.1)	2 (1.3)	3 (2.0)
Number of subjects with comorbidities, n (%)	33 (100%)	15 (100%)	48 (100%)
Hypertension	11 (33.3%)	6 (40%)	17 (35.4%)
Diabetes mellitus type II	5 (15.2%)	3 (20%)	8 (16.7%)
Diabetes mellitus	3 (9.1%)	3 (20%)	6 (12.5%)
None	3 (9.1%)	3 (20%)	6 (12.5%)
Hyperlipidemia	5 (15.2%)	0 (0%)	5 (10.4%)
Obesity	4 (12.1%)	1 (6.7%)	5 (10.4%)
(For complete list, see Supplementary material)			
Concomitant medications, *n* (%)			
Number of medications per subject, M (SD)	24.7 (15.9)	17.2 (13.7)	22.4 (15.5)
Number of subjects with concomitant medication	32 (97%)	14 (93.3%)	46 (95.8%)
Zinc Sulfate	29 (87.9%)	11 (73.3%)	40 (83.3%)
Atorvastatin	26 (78.8%)	10 (66.7%)	36 (75%)
Famotidine	24 (72.7%)	10 (66.7%)	34 (70.8%)
Melatonin	25 (75.8%)	9 (60%)	34 (70.8%)
Methylprednisolone	25 (75.8%)	8 (53.3%)	33 (68.8%)
Hydroxychloroquine	23 (69.7%)	9 (60%)	32 (66.7%)
Ivermectin	21 (63.6%)	7 (46.7%)	28 (58.3%)
Enoxaparin	19 (57.6%)	8 (53.3%)	27 (56.3%)
Ascorbic Acid 3,000 mg/NS	17 (51.5%)	9 (60%)	26 (54.2%)
Cholecalciferol	19 (57.6%)	7 (46.7%)	26 (54.2%)
Parenteral Electrolytes	17 (51.5%)	9 (60%)	26 (54.2%)
Thiamine 200 mg/D5W	15 (45.5%)	6 (40%)	21 (43.8%)
Magnesium Sulfate 2 gm/NS	14 (42.4%)	6 (40%)	20 (41.7%)
(For complete list, see Supplementary material)			

HB-adMSCs 100MM, allogeneic Hope Biosciences adipose-derived MSCs 1×10^8^ cells. Comorbidities with 5 or more occurrences and medications with 20 or more occurrences in the overall sample are listed in the table. For full list of medications and comorbidities by group, see Supplementary Table S1.

The trial was placed on clinical hold on August 6, 2020, due to a notable increase in the number of SAEs, including deaths, reported to the FDA. Following comprehensive investigations that determined none of the reported deaths could be attributed to the investigational drug, the FDA lifted the clinical hold on April 23, 2021. However, by that point in time, COVID-19 vaccines had become widely accessible, and the pandemic wave had significantly receded. Consequently, the study sponsor opted to discontinue the trial.

### 3.2 Safety

#### 3.2.1 Adverse events

A total of 119 AEs were recorded during the study period, out of which 105 were mild in severity, 2 moderate, 1 severe, 1 life threatening, and 10 fatal (death). Twelve total SAEs were reported during the study period. The most frequent AEs were body aches, dyspnea, cardiopulmonary failure, cough, headache, hyperthermia, fatigue, anxiety, hypertension, anemia, chest discomfort, chills, and diarrhea. Analyses showed higher incidence of cardiopulmonary failure (relative risk (RR) = 1.59, PP = 80.0%), anemia (RR = 1.59, PP = 95.0%), anxiety (RR = 12.74, PP = 95.0%) and diarrhea (RR = 11.20, PP = 93.0%) in HB-adMSC versus placebo. Placebo showed a higher incidence of headaches (RR = 0.32, PP = 92.0%), fatigue (RR = 0.26, PP = 96.0%), and chest discomfort (RR = 0.30, PP = 87.0%) compared to HB-adMSC.

Deaths that occurred during the treatment period were solely due to severe complications from COVID-19 and were unrelated to the study drug (Table 3). All reported AEs were considered either unlikely or unrelated to the investigational product; none were attributed to the investigational product. Most reported AEs (109 of 119) were identified as recovered without sequelae (Supplementary Table S2).

**Table 3:** Adverse events by group.

Characteristic	HB-adMSCs 100MM, *N* = 33		Placebo, *N* = 15		Overall, *N* = 48	
	N (%)	Events	N (%)	Events	N (%)	Events
Adverse events	33	80	15	39	48	119
Adverse event, n (%)						
Body Aches	7 (21.2%)	9	4 (26.7%)	5	11 (22.9%)	14
Dyspnea	7 (21.2%)	7	2 (13.3%)	2	9 (18.8%)	9
Cardiopulmonary failure	6 (18.2%)	6	2 (13.3%)	2	8 (16.7%)	8
Cough	5 (15.2%)	5	3 (20.0%)	3	8 (16.7%)	8
Headache	2 (6.1%)	4	3 (20.0%)	4	5 (10.4%)	4
Hyperthermia	4 (12.1%)	6	2 (13.3%)	2	6 (12.5%)	8
Fatigue	2 (6.1%)	2	4 (26.7%)	4	6 (12.5%)	6
(For complete list, see Supplementary material)						
Adverse event severity, n (%)						
Mild	25 (75.8%)	68	12 (80.0%)	37	37 (77.1%)	105
Moderate	2 (6.1%)	2	0 (0.0%)		2 (4.2%)	2
Severe	1 (3.0%)	1	0 (0.0%)		1 (2.1%)	1
Life-threatening	1 (3.0%)	1	0 (0.0%)		1 (2.1%)	1
Fatal	8 (24.2%)	8	2 (13.3%)	2	10 (20.8%)	10
Attribution to study drug, n (%)						
Definite						
Probable						
Possible						
Unlikely	8 (24.2%)	18	8 (53.3%)	15	16 (33.3%)	33
Unrelated	24 (72.7%)	62	11 (73.3%)	24	35 (72.9%)	86
Serious, n (%)	9 (26.5%)	10	2 (14.3%)	2	11 (22.9%)	12

HB-adMSCs 100MM, allogeneic Hope Biosciences adipose-derived MSCs 1×10^8^ cells. Adverse event types with 5 or more occurrences are listed in the table. For full list of adverse events and serious adverse events, see Supplementary Table S2.

#### 3.2.2 Laboratory assessments

Comparisons of the standard laboratory parameters (hematologic, biochemistry, and coagulation panel) at baseline and end of study showed no significant differences between the two groups at any timepoint. There were no significant changes in the laboratory parameters when compared to the baseline. Evaluation of laboratory measures, vital signs, weight, and physical examination also showed no clinically significant changes (Supplementary Tables S3–S5).

##### 3.2.2.1 Coagulation parameters

The moderating influence of group on time-varying effects on coagulation parameters were evaluated to assess safety and tolerability. No differential change over time by group was found in WBC, platelets, neutrophils, PT, or PTT (Table 4). Differential change was found in lymphocytes (Time x Group interaction *p* = 0.017), such that placebo showed an increase over time (*b* = 5.07, *p* < 0.001) compared to HB-adMSC (*b* = 1.36, *p* = 0.071). Though there was an overall difference between groups in prothrombin INR levels across time (*p* = 0.032, HB-adMSC mean_marginal_ = 1.2, placebo mean_marginal_ = 1.0), there was no differential change over time.

**Table 4:** Coagulation parameters.

	Infusion 1 (Baseline)			Infusion 2			Infusion 3			Infusion 4			Regression result	
Characteristic, M (SD)	N	HB-adMSCs100MM, *N* = 30	Placebo, *N* = 13	N	HB-adMSCs100MM, *N* = 24	Placebo, *N* = 11	N	HB-adMSCs100MM, *N* = 18	Placebo, *N* = 9	N	HB-adMSCs 100MM, *N* = 17	Placebo, *N* = 9	Time x Group interaction *p*	Group main effect *p*
WBC (x10^3^/uL)	42	11.3 (10.5)	10.3 (3.6)	34	13.5 (11.3)	13.8 (8.7)	27	12.1 (5.8)	12.7 (5.5)	23	10.4 (5.0)	11.4 (5.8)	0.624	0.787
Lymphocytes (%)	42	11.3 (8.2)	12.1 (7.9)	33	14.5 (13.9)	17.2 (12.7)	27	17.4 (13.3)	16.8 (13.5)	21	17.0 (11.8)	25.7 (14.2)	**0.017**	0.542
Platelets (x10^3^/uL)	40	237.5 (98.8)	304.8 (101.1)	34	258.2 (115.7)	295.8 (109.0)	26	273.1 (119.6)	323.1 (101.2)	22	245.2 (110.5)	303.8 (86.5)	0.936	0.161
Neutrophils (%)	42	79.4 (15.4)	80.7 (11.3)	33	77.0 (15.3)	75.3 (15.9)	27	73.5 (15.6)	75.7 (16.6)	21	75.1 (13.3)	66.2 (17.2)	0.056	0.959
Prothrombin INR	36	1.2 (0.3)	1.1 (0.1)	29	1.2 (0.3)	1.1 (0.1)	26	1.2 (0.4)	1.1 (0.1)	21	1.4 (0.6)	1.1 (0.2)	0.573	**0.032**
PT (sec)	36	14.3 (3.9)	12.9 (2.0)	29	14.4 (3.1)	13.9 (2.0)	26	15.3 (4.1)	14.6 (1.4)	21	16.1 (5.8)	14.3 (1.7)	0.878	0.192
PTT (sec)	18	31.6 (6.6)	29.8 (4.2)	14	32.2 (5.8)	26.5 (6.4)	4	31.0 (6.9)	NA	8	24.5 (12.7)	28.2 (1.4)	0.567	0.076

HB-adMSCs 100MM, allogeneic Hope Biosciences adipose-derived MSCs 1×10^8^ cells. TNF-α, Tumor Necrosis Factor-alpha. CRP, C-Reactive Protein. For coagulation parameters (safety and tolerability), regression results (*p* values) were derived from frequentist models evaluating changes over time (infusions 1 through 4) in each group. Boldface p-values indicated *p* <0.05.

##### 3.2.2.2 Inflammatory markers

Time-varying effects on TNF-α, interleukin-10, interleukin-6, and CRP were evaluated using Bayesian inference to assess efficacy. Results showed modest differential changes in most markers over time, signified by Time x Group interaction PP ≥ 75%. HB-adMSC exhibited greater reductions in TNF-α and interleukin-10, but increased levels of interleukin-6 and CRP over time, compared to placebo (Table 5).

**Table 5:** Inflammatory markers.

	Infusion 1 (Baseline)			Infusion 2			Infusion 3			Infusion 4			Regression result	
Characteristic, M (SD)	N	HB-adMSCs100MM, *N* = 30	Placebo, *N* = 13	N	HB-adMSCs 100MM, *N* = 24	Placebo, *N* = 11	N	HB-adMSCs 100MM, *N* = 18	Placebo, *N* = 9	N	HB-adMSCs 100MM, *N* = 17	Placebo, *N* = 9	Time × Group interaction PP	Group main effect PP
TNF-α, M (SD)	25	15.5 (13.9)	18.2 (12.3)	19	26.1 (26.5)	15.9 (10.0)	29	33.4 (49.8)	35.3 (38.8)	24	20.3 (11.3)	30.5 (40.5)	**82.6%**	57.4%
Interleukin-10, M (SD)	23	16.3 (19.4)	11.9 (16.6)	19	18.8 (39.1)	44.7 (98.9)	26	30.8 (86.3)	27.2 (65.1)	13	13.0 (13.5)	8.4 (10.8)	**83.9%**	**91.4%**
Interleukin-6, M (SD)	39	24.3 (71.8)	69.4 (166.5)	29	46.3 (104.0)	376.6 (988.7)	28	65.4 (186.8)	197.8 (539.7)	26	35.0 (70.4)	12.4 (23.9)	**86.3%**	52%
CRP, M (SD)	37	4.9 (5.8)	3.8 (5.1)	29	4.8 (7.5)	4.5 (9.2)	21	3.6 (3.4)	2.2 (4.1)	24	3.5 (4.8)	0.2 (0.2)	**78.8%**	**99.5%**

HB-adMSCs 100MM, allogeneic Hope Biosciences adipose-derived MSCs 1×10^8^ cells. TNF-α, Tumor Necrosis Factor-alpha. CRP, C-Reactive Protein. PP, posterior probability. For inflammatory markers (efficacy), regression results (posterior probabilities) were derived from Bayesian models evaluating changes over time (infusions 1 through 4) in each group (see Supplementary Data in Supplementary material for descriptions of Bayesian methods). Boldface PP values indicated PP ≥ 75%.

## 4 Discussion

The main purpose of the current study was to evaluate the safety of allogeneic MSCs in hospitalized subjects with COVID-19 symptoms. Results showed that multiple infusions of allogeneic HB-adMSCs were safe and well tolerated by participants, with little-to-no changes in coagulation parameters in the treatment group versus placebo, and no attribution of any reported AEs to the study drug.

Although randomization was performed to prevent enrollment bias, an imbalance emerged between groups at baseline, with more high-risk pre-existing medical conditions and number of concomitant medications, with a higher proportion of both in the HB-adMSC group. This imbalance potentially conferred greater vulnerability in the HB-adMSC subjects to COVID-19-related complications, as shown by the overall more frequent AEs in the HB-adMSC group. This finding is also consistent with another study that found higher COVID-19-related mortality associated with multiple comorbidities (16). Despite the higher incidence of specific AEs in the HB-adMSC group, the majority of AEs were mild in severity, and none were attributed to the study drug. Deaths that occurred during the study period were solely due to severe complications from COVID-19 and were unrelated to the study drug.

Safety findings in the current study are consistent with previous reports on the safety of MSCs for the treatment of COVID-19 (12–15, 17–21). These reports include the earliest randomized MSC trials in 2020 in patients with moderate to severe COVID-19 pneumonia, finding no acute adverse events following single or multiple infusions of umbilical cord-derived MSCs (17–19). Similar safety findings were reported in two early non-randomized trials performed in patients with severe COVID-19 or critically ill status, one in a single-group pre-post design using a single infusion of umbilical cord-derived MSCs (20) and another comparing treatment-as-usual (concomitant medications) versus a comprehensive regimen (concomitant medications and three infusions of menstrual blood-derived MSCs) (21). In all aforementioned trials, AEs and SAEs were considered not related to the infusions, and any AEs were frequently attributed progression to more severe disease. Deaths, where reported, were also deemed not to be related to MSC infusion (15, 20, 21). In one randomized trial that compared two infusions of umbilical cord derived MSCs versus placebo in patients with mild to severe COVID-19 ARDS, there were significantly more SAEs and deaths in the placebo versus the treatment group (13). All deaths were deemed unrelated to infusions, and most were considered to be related to acute respiratory failure or multi-organ dysfunction syndrome. In another randomized trial that compared multiple infusions of umbilical cord derived MSCs versus placebo in patients with mild to moderate COVID-19 related ARDS, headache was the most frequent AE, and deaths in the treatment group were solely due to progression to more severe ARDS (12). Finally, two more recent randomized trials in patients with severe COVID-19 found similar safety profiles as the aforementioned trials that enrolled patients with less severe illness (14, 15). A trial in 20 critically ill intensive care unit patients in the last stages of COVID-19 related pneumonia found that a single infusion of placenta-derived MSCs was safe and tolerable; both the MSC and placebo groups saw 50% deaths that were all unrelated to MSCs (15). A study that retrospectively compared three infusions of bone-marrow derived MSCs in 8 intensive care unit patients with 24 matched controls found no adverse events related to MSC infusions (14). Given most aforementioned studies focused on patients with severe COVID-19 pneumonia or ARDS, the safety profile in the current study is in line with what has been reported. Considering especially the trials that enrolled patients with mild or moderate COVID-19 symptoms (12, 13), the safety findings in the current study may translate to lower overall incidence of and less severe AEs in populations with comparatively lower severity of illness.

Imbalances in comorbidities and medications in the HB-adMSC group may explain the lack of efficacy on inflammatory markers. The HB-adMSC group showed reduced TNF-α and interleukin-10, along with elevated interleukin-6 and CRP, consistent with excessive COVID-19-like immune response that involves elevations in proinflammatory cytokines such as TNF-α, interleukin-6, interleukin-10, and interferon-γ (22–25). These suggest that the treatment group, via its baseline vulnerability, had worse prognosis potentially due to the cytokine storm implicated in the immunomodulatory response to COVID-19. The overall lack of efficacy suggests MSC therapy was not efficacious in rescuing critically ill COVID-19 patients with severe ARDS. This is consistent with previous trials that found no clinical effects in patients with ARDS (26, 27), warranting more research into MSC therapy efficacy for moderate-to-severe COVID-19 symptoms.

Despite the baseline imbalance of preexisting conditions and medications between groups, all subjects received standard-of-care treatment. All subjects in both placebo and treatment groups had comorbidities at baseline, with a greater number of high-risk conditions in the treatment group. Most subjects (96%) entered the study with at least one concomitant medication. Given these facts and the lack of available established treatments for COVID-19 at the start of the trial (June 2020), adherence to study protocol and standard of care was crucial.

The trial was not without limitations. Imbalance in baseline comorbidities may have influenced rates of AEs. Further, due to imbalances in the group sizes and observed under-enrollment, estimates may not be as robust. Thus, further research with larger sample sizes and better-balanced groups is needed. In addition, most study subjects were crucially ill, therefore findings in the current trial may not be generalized to patients with milder symptoms or fewer comorbidities.

Despite the lack of observed efficacy, multiple intravenous infusions of HB-adMSCs were safe and tolerable. There was no risk of thrombi formation, suggesting treatment did not pose any thrombogenic risk. Given the low survival rate in critically-ill COVID-19 patients (28) and the complexity of the disease, using multiple treatment strategies including MSC therapies may potentially increase survival.

## Data availability statement

The original contributions presented in the study are included in the article/Supplementary material, further inquiries can be directed to the corresponding author.

## Ethics statement

The studies involving humans were approved by Western International Review Board, Inc. Washington, USA. The studies were conducted in accordance with the local legislation and institutional requirements. The participants provided their written informed consent to participate in this study.

## Author contributions

CD: Formal analysis, Writing – original draft, Writing – review & editing, Data curation. RV: Conceptualization, Data curation, Formal analysis, Writing – original draft, Writing – review & editing. HK: Resources, Writing – review & editing. HP: Resources, Writing – review & editing. DC: Funding acquisition, Supervision, Writing – review & editing.
