# Supplementary material for: Safety of multiple intravenous infusions of adipose-derived mesenchymal stem cells for hospitalized cases of COVID-19: a randomized controlled trial
Source: Front Med (Lausanne). 2023 Dec 22;10:1321303. doi: 10.3389/fmed.2023.1321303 (PMC10770855; doi:10.3389/fmed.2023.1321303)
Supplement: Supplementary file 1 [file Data_Sheet_1.docx]

Supplementary Material

# Supplementary Data

## Bayesian inference: General Considerations

The analytical approach for inflammatory markers used Bayesian statistical methods to assess the probability that an effect of HB-adMSCs 100mm exists (relative to placebo). Our analytical plan was shaped by the limitations of conventional (Frequentist) methods for addressing this question and the advantages of a Bayesian approach for assessing the probability that a given strategy might successfully be expanded into a larger-scale program for the treatment of COVID-19 symptoms. This data, valuable in its own right, can justify the commitment of resources needed for such an expansion. Further, current uncertainty regarding the probability of worse status on the inflammatory markers as a function of HB-adMSC 100mm was readily incorporated in a Bayesian approach permitting more robust trial planning and design.

The Bayesian approach addresses these questions: (1) “Among patients with suspected COVID-19, what is the probability that allogeneic HB-adMSC 100mm confers benefit relative to placebo on status on inflammatory markers (C-Reactive protein, TNF-alpha, IL-6, IL-10) at treatment Day 10?” (2) “What is the best estimate of these effects?” and (3) “What is their precision?” By estimating the probability that such effects exist, we assessed the probability that the alternative hypothesis was true; a probability that is, by definition, not accessible to Frequentist methods. The FDA has discussed the use of Bayesian statistical methods to make decisions regarding the efficacy of new treatments as an alternative to Frequentist methods in developing clinical applications (1,2). The current proposal provided the optimal, unbiased estimates for the benefit conferred by allogeneic HB-adMSC, while also estimating the probability that such effects exist.

Presentation of results from the statistical models included the estimated conditional/marginal mean treatment effects. For all endpoints analysed statistically, estimated mean treatment differences were presented together with 95% credible intervals (95% CrI) and posterior probabilities (PP). For the purposes of evaluating the comparability of groups, PP≥75% constituted evidence for statistically reliable differences.

## Statistical Methodology Specification

Analyses of inflammatory markers and specific adverse events used generalized linear models (R v 4.1, 2021; brms v 2.17; rstan v 2.21) for continuous and dichotomous outcomes (3–5). Multilevel generalized linear modeling was used to account for clustering of patients within site and repeated observations within patients. All analyses addressed potential missingness through joint modeling of observed outcomes and the missing data, an approach robust to ignorable missingness (i.e., MCAR and MAR) (6).

Evaluation of posterior distributions permitted statements regarding the probability that effects of varying magnitudes exist, given the data. Specification of diffuse, neutral priors reflected the initial uncertainty regarding effect sizes. For all generalized linear models, priors for regression coefficients were be specified as ~Normal (μ=0, σ^2^=1 x 10), and priors for the levels one and two error variances were specified as ~Half- Normal (μ=0, σ^2^=1 x 10^3^). The choice of prior distribution for level two variances followed Gelman’s recommendations from the literature (7).

**Inflammatory markers.** All primary endpoint efficacy measurements available at post-baseline at scheduled measurements (Visits 2 through 5 [infusions 1 through 4] for inflammatory markers) were analyzed in a generalized linear mixed model. The model predicted each outcome as a function of the interaction between the fixed factors treatment group and time, controlling for lower order effects of time and treatment group. Models controlled for stratification variables, and subject was included as random effects.

**Incidence of specific adverse events**. Specific adverse events were analyzed in a generalized linear model via the binomial distribution using a log-link. The model predicted the presence of a given adverse event as a function of treatment group. Models controlled for stratification variables.

# Supplementary Tables

Supplementary Table 1. Full table of medical history and concomitant medications.

| **Characteristic** | **HB-adMSCs 100MM**, N = 33 | **Placebo**, N = 15 | **Overall**, N = 48 |
| --- | --- | --- | --- |
|  |  |  |  |
| Number of subjects with comorbidities, n (%) | 33 (100) | 15 (100) | 48 (100) |
| Hypertension | 11 (33.3%) | 6 (40%) | 17 (35.4%) |
| Diabetes Mellitus Type II | 5 (15.2%) | 3 (20%) | 8 (16.7%) |
| Diabetes Mellitus | 3 (9.1%) | 3 (20%) | 6 (12.5%) |
| None | 3 (9.1%) | 3 (20%) | 6 (12.5%) |
| Hyperlipidemia | 5 (15.2%) | 0 (0%) | 5 (10.4%) |
| Obesity | 4 (12.1%) | 1 (6.7%) | 5 (10.4%) |
| Asthma | 3 (9.1%) | 1 (6.7%) | 4 (8.3%) |
| Hypothyroidism | 3 (9.1%) | 1 (6.7%) | 4 (8.3%) |
| Coronary Artery Disease | 3 (9.1%) | 0 (0%) | 3 (6.3%) |
| Depression | 2 (6.1%) | 1 (6.7%) | 3 (6.3%) |
| Gastroesophageal reflux disease | 2 (6.1%) | 1 (6.7%) | 3 (6.3%) |
| Osteoarthritis | 2 (6.1%) | 1 (6.7%) | 3 (6.3%) |
| Anxiety | 1 (3%) | 1 (6.7%) | 2 (4.2%) |
| Atrial fibrillation | 2 (6.1%) | 0 (0%) | 2 (4.2%) |
| Dementia | 1 (3%) | 1 (6.7%) | 2 (4.2%) |
| Diabetes Mellitus Type I | 2 (6.1%) | 0 (0%) | 2 (4.2%) |
| DVT | 2 (6.1%) | 0 (0%) | 2 (4.2%) |
| Ex-smoker | 2 (6.1%) | 0 (0%) | 2 (4.2%) |
| Ex-Smoker | 2 (6.1%) | 0 (0%) | 2 (4.2%) |
| Gastritis | 2 (6.1%) | 0 (0%) | 2 (4.2%) |
| Gout | 1 (3%) | 1 (6.7%) | 2 (4.2%) |
| Left Hemiparesis | 2 (6.1%) | 0 (0%) | 2 (4.2%) |
| Primary (essential) Hypertension | 2 (6.1%) | 0 (0%) | 2 (4.2%) |
| Vertigo | 2 (6.1%) | 0 (0%) | 2 (4.2%) |
| ADHD | 1 (3%) | 0 (0%) | 1 (2.1%) |
| Anemia | 1 (3%) | 0 (0%) | 1 (2.1%) |
| Arthralgia | 1 (3%) | 0 (0%) | 1 (2.1%) |
| Atrial Fibrillation | 1 (3%) | 0 (0%) | 1 (2.1%) |
| Attention Deficit Disorder (ADD) | 1 (3%) | 0 (0%) | 1 (2.1%) |
| Benign Prostatic Hypertrophy | 1 (3%) | 0 (0%) | 1 (2.1%) |
| Carotid Stenosis | 1 (3%) | 0 (0%) | 1 (2.1%) |
| Cerebral Vascular Accident | 1 (3%) | 0 (0%) | 1 (2.1%) |
| Chronic Kidney Disease (CKD) | 1 (3%) | 0 (0%) | 1 (2.1%) |
| Chronic Kidney Disease Stage III | 1 (3%) | 0 (0%) | 1 (2.1%) |
| Chronic Obstructive Pulmonary Disease (COPD) | 1 (3%) | 0 (0%) | 1 (2.1%) |
| Congestive Heart Failure | 1 (3%) | 0 (0%) | 1 (2.1%) |
| Congestive Heart Failure (CHF) | 1 (3%) | 0 (0%) | 1 (2.1%) |
| Coronary Artery Disease (CAD) | 1 (3%) | 0 (0%) | 1 (2.1%) |
| Degenerative Cervical Disc Disease | 1 (3%) | 0 (0%) | 1 (2.1%) |
| Diabetes Mellitus (uncontrolled) | 1 (3%) | 0 (0%) | 1 (2.1%) |
| Dyslipidemia | 0 (0%) | 1 (6.7%) | 1 (2.1%) |
| Encephalopathy | 1 (3%) | 0 (0%) | 1 (2.1%) |
| End Stage Renal Disease-Hemodialysis | 1 (3%) | 0 (0%) | 1 (2.1%) |
| Eye disease | 1 (3%) | 0 (0%) | 1 (2.1%) |
| Fatty Liver Disease | 1 (3%) | 0 (0%) | 1 (2.1%) |
| Gastric Esophageal Reflux Disease | 0 (0%) | 1 (6.7%) | 1 (2.1%) |
| Glaucoma | 1 (3%) | 0 (0%) | 1 (2.1%) |
| H/O Congestive Heart Failure | 1 (3%) | 0 (0%) | 1 (2.1%) |
| H/O Diabetes Insulin Dependent | 1 (3%) | 0 (0%) | 1 (2.1%) |
| H/O Hypertension | 1 (3%) | 0 (0%) | 1 (2.1%) |
| H/O Liver Cancer | 0 (0%) | 1 (6.7%) | 1 (2.1%) |
| H/O Peripheral Vascular Disease | 0 (0%) | 1 (6.7%) | 1 (2.1%) |
| Hepatic Lesion | 1 (3%) | 0 (0%) | 1 (2.1%) |
| Hepatitis B | 1 (3%) | 0 (0%) | 1 (2.1%) |
| HIV | 1 (3%) | 0 (0%) | 1 (2.1%) |
| Hypercholesteremia | 0 (0%) | 1 (6.7%) | 1 (2.1%) |
| Hypercholesterolemia | 1 (3%) | 0 (0%) | 1 (2.1%) |
| Illicit Drug Abuse | 1 (3%) | 0 (0%) | 1 (2.1%) |
| Insulin dependent diabetes mellitus | 1 (3%) | 0 (0%) | 1 (2.1%) |
| Insulin Dependent Diabetes Mellitus | 1 (3%) | 0 (0%) | 1 (2.1%) |
| Left Hemiplegia | 1 (3%) | 0 (0%) | 1 (2.1%) |
| Migraine | 1 (3%) | 0 (0%) | 1 (2.1%) |
| Muscle weakness | 0 (0%) | 1 (6.7%) | 1 (2.1%) |
| Myalgia | 1 (3%) | 0 (0%) | 1 (2.1%) |
| Osteoporosis | 1 (3%) | 0 (0%) | 1 (2.1%) |
| Parkinson's Disease | 1 (3%) | 0 (0%) | 1 (2.1%) |
| Peripheral Artery Disease | 1 (3%) | 0 (0%) | 1 (2.1%) |
| Peripheral Vascular Disease (PVD) | 1 (3%) | 0 (0%) | 1 (2.1%) |
| Polycystic ovarian Syndrome | 1 (3%) | 0 (0%) | 1 (2.1%) |
| Prediabetes | 1 (3%) | 0 (0%) | 1 (2.1%) |
| Radiculapathy | 1 (3%) | 0 (0%) | 1 (2.1%) |
| Smoker | 1 (3%) | 0 (0%) | 1 (2.1%) |
| Stroke (10 years ago) | 1 (3%) | 0 (0%) | 1 (2.1%) |
| Thrombocytopenia | 1 (3%) | 0 (0%) | 1 (2.1%) |
| Urinary tract infection (UTI) | 1 (3%) | 0 (0%) | 1 (2.1%) |
| Vitamin D deficiency | 1 (3%) | 0 (0%) | 1 (2.1%) |
| Vitamin D Low | 1 (3%) | 0 (0%) | 1 (2.1%) |
|  |  |  |  |
| Number of medications per subject, M (SD) | 24.7 (15.9) | 17.2 (13.7) | 22.4 (15.5) |
| Number of subjects with concomitant medication, n (%) | 32 (97%) | 14 (93.3%) | 46 (95.8%) |
| Zinc Sulfate | 29 (87.9%) | 11 (73.3%) | 40 (83.3%) |
| Atorvastatin | 26 (78.8%) | 10 (66.7%) | 36 (75%) |
| Famotidine | 24 (72.7%) | 10 (66.7%) | 34 (70.8%) |
| Melatonin | 25 (75.8%) | 9 (60%) | 34 (70.8%) |
| Methylprednisolone | 25 (75.8%) | 8 (53.3%) | 33 (68.8%) |
| Hydroxychloroquine | 23 (69.7%) | 9 (60%) | 32 (66.7%) |
| Ivermectin | 21 (63.6%) | 7 (46.7%) | 28 (58.3%) |
| Enoxaparin | 19 (57.6%) | 8 (53.3%) | 27 (56.3%) |
| Ascorbic Acid 3000mg/NS | 17 (51.5%) | 9 (60%) | 26 (54.2%) |
| Cholecalciferol | 19 (57.6%) | 7 (46.7%) | 26 (54.2%) |
| Parenteral Electrolytes | 17 (51.5%) | 9 (60%) | 26 (54.2%) |
| Thiamine 200mg/D5W | 15 (45.5%) | 6 (40%) | 21 (43.8%) |
| Magnesium Sulfate 2 gm/NS | 14 (42.4%) | 6 (40%) | 20 (41.7%) |
| Insulin Human Regular | 16 (48.5%) | 3 (20%) | 19 (39.6%) |
| Azithromycin | 11 (33.3%) | 3 (20%) | 14 (29.2%) |
| Lisinopril | 10 (30.3%) | 4 (26.7%) | 14 (29.2%) |
| Colchicine | 9 (27.3%) | 2 (13.3%) | 11 (22.9%) |
| Itraconazole | 9 (27.3%) | 1 (6.7%) | 10 (20.8%) |
| Morphine Sulfate | 9 (27.3%) | 1 (6.7%) | 10 (20.8%) |
| Nelfinavir | 6 (18.2%) | 4 (26.7%) | 10 (20.8%) |
| Azithromycin 250mg/NS | 5 (15.2%) | 4 (26.7%) | 9 (18.8%) |
| Ascorbic Acid | 7 (21.2%) | 1 (6.7%) | 8 (16.7%) |
| Tylenol | 5 (15.2%) | 3 (20%) | 8 (16.7%) |
| Fluconazole | 6 (18.2%) | 1 (6.7%) | 7 (14.6%) |
| Furosemide | 6 (18.2%) | 1 (6.7%) | 7 (14.6%) |
| hydroxychloroquine | 4 (12.1%) | 3 (20%) | 7 (14.6%) |
| Lorazepam | 7 (21.2%) | 0 (0%) | 7 (14.6%) |
| Mycophenolate Mofetil | 5 (15.2%) | 2 (13.3%) | 7 (14.6%) |
| Thiamine | 6 (18.2%) | 1 (6.7%) | 7 (14.6%) |
| Azithromycin 250 mg/NS | 3 (9.1%) | 3 (20%) | 6 (12.5%) |
| Azithromycin 500mg/NS | 3 (9.1%) | 3 (20%) | 6 (12.5%) |
| cholecalciferol | 5 (15.2%) | 1 (6.7%) | 6 (12.5%) |
| Acetaminophen | 5 (15.2%) | 0 (0%) | 5 (10.4%) |
| Lactulose | 4 (12.1%) | 1 (6.7%) | 5 (10.4%) |
| Methylprednisolone (Solu-Medrol) | 3 (9.1%) | 2 (13.3%) | 5 (10.4%) |
| Methylprednisolone 1000 mg/NS | 4 (12.1%) | 1 (6.7%) | 5 (10.4%) |
| Nelfinavir (Viracept) | 3 (9.1%) | 2 (13.3%) | 5 (10.4%) |
| Nelfinavir Mesylate (Viracept) | 4 (12.1%) | 1 (6.7%) | 5 (10.4%) |
| Potassium Chloride | 4 (12.1%) | 1 (6.7%) | 5 (10.4%) |
| Amlodipine | 2 (6.1%) | 2 (13.3%) | 4 (8.3%) |
| Azithromycin 500 mg/NS | 3 (9.1%) | 1 (6.7%) | 4 (8.3%) |
| Clonidine | 3 (9.1%) | 1 (6.7%) | 4 (8.3%) |
| Enoxaparin (Lovenox) | 2 (6.1%) | 2 (13.3%) | 4 (8.3%) |
| Furosemide (Lasix) | 3 (9.1%) | 1 (6.7%) | 4 (8.3%) |
| methylprednisolone | 3 (9.1%) | 1 (6.7%) | 4 (8.3%) |
| Methylprednisolone Sodium Succinate | 2 (6.1%) | 2 (13.3%) | 4 (8.3%) |
| Methylprednisolone Sodium Succinate (Solu-Medrol) | 3 (9.1%) | 1 (6.7%) | 4 (8.3%) |
| Tocilizumab | 3 (9.1%) | 1 (6.7%) | 4 (8.3%) |
| TPN | 2 (6.1%) | 2 (13.3%) | 4 (8.3%) |
| Trimethoprim/Sulfamethoxazole | 3 (9.1%) | 1 (6.7%) | 4 (8.3%) |
| Zolpidem | 3 (9.1%) | 1 (6.7%) | 4 (8.3%) |
| Albumin Human | 3 (9.1%) | 0 (0%) | 3 (6.3%) |
| Ascorbic Acid 3000 mg/NS | 2 (6.1%) | 1 (6.7%) | 3 (6.3%) |
| Cholecalciferol (Vit. D3) | 1 (3%) | 2 (13.3%) | 3 (6.3%) |
| Doxycycline | 3 (9.1%) | 0 (0%) | 3 (6.3%) |
| Enteral Nutritional Formula | 2 (6.1%) | 1 (6.7%) | 3 (6.3%) |
| Ertapenem Sodium 1 gm/NS | 2 (6.1%) | 1 (6.7%) | 3 (6.3%) |
| Fat Emulsion | 1 (3%) | 2 (13.3%) | 3 (6.3%) |
| Hydroxychloroquine Sulfate | 1 (3%) | 2 (13.3%) | 3 (6.3%) |
| Mag Sulfate | 3 (9.1%) | 0 (0%) | 3 (6.3%) |
| Magnesium Sulfate | 1 (3%) | 2 (13.3%) | 3 (6.3%) |
| Magnesium Sulfate/Dextrose | 2 (6.1%) | 1 (6.7%) | 3 (6.3%) |
| Meropenem | 3 (9.1%) | 0 (0%) | 3 (6.3%) |
| Norepinephrine Bitartrate 8 mg/D5W | 3 (9.1%) | 0 (0%) | 3 (6.3%) |
| Ondansetron | 2 (6.1%) | 1 (6.7%) | 3 (6.3%) |
| parenteral electrolytes | 2 (6.1%) | 1 (6.7%) | 3 (6.3%) |
| Plasmalyte | 3 (9.1%) | 0 (0%) | 3 (6.3%) |
| Remdesivir 100mg/NS | 2 (6.1%) | 1 (6.7%) | 3 (6.3%) |
| Remdesivir 200 mg/NS | 2 (6.1%) | 1 (6.7%) | 3 (6.3%) |
| Sodium Bicarbonate | 3 (9.1%) | 0 (0%) | 3 (6.3%) |
| Sodium Chloride | 3 (9.1%) | 0 (0%) | 3 (6.3%) |
| Thiamine 200mg/NS | 2 (6.1%) | 1 (6.7%) | 3 (6.3%) |
| Tramadol | 1 (3%) | 2 (13.3%) | 3 (6.3%) |
| Vasopressin 40 units/NS | 3 (9.1%) | 0 (0%) | 3 (6.3%) |
| Zolpidem Tartrate | 3 (9.1%) | 0 (0%) | 3 (6.3%) |
| Ceftriaxone 2 gm/NS | 2 (6.1%) | 0 (0%) | 2 (4.2%) |
| Celecoxib | 2 (6.1%) | 0 (0%) | 2 (4.2%) |
| Clopidogrel Bisulfate | 2 (6.1%) | 0 (0%) | 2 (4.2%) |
| Dextrose 50% | 2 (6.1%) | 0 (0%) | 2 (4.2%) |
| Doxycycline 100mg/NS | 1 (3%) | 1 (6.7%) | 2 (4.2%) |
| Doxycycline Hyclate 100 mg/NS | 1 (3%) | 1 (6.7%) | 2 (4.2%) |
| Eliquis | 2 (6.1%) | 0 (0%) | 2 (4.2%) |
| Enoxaparin Sodium (Lovenox) | 2 (6.1%) | 0 (0%) | 2 (4.2%) |
| famotidine | 1 (3%) | 1 (6.7%) | 2 (4.2%) |
| Filgrastim | 2 (6.1%) | 0 (0%) | 2 (4.2%) |
| Hydralazine | 2 (6.1%) | 0 (0%) | 2 (4.2%) |
| Hydromorphone (Dilaudid) | 2 (6.1%) | 0 (0%) | 2 (4.2%) |
| Insulin Human Regualar | 1 (3%) | 1 (6.7%) | 2 (4.2%) |
| Ivermectin | 2 (6.1%) | 0 (0%) | 2 (4.2%) |
| Labetalol | 1 (3%) | 1 (6.7%) | 2 (4.2%) |
| Lactulose Syrup | 2 (6.1%) | 0 (0%) | 2 (4.2%) |
| Levothyroxine | 2 (6.1%) | 0 (0%) | 2 (4.2%) |
| Lorazepam (Ativan) | 2 (6.1%) | 0 (0%) | 2 (4.2%) |
| Magnesium Sulfate 2 gm/D5W | 1 (3%) | 1 (6.7%) | 2 (4.2%) |
| Metformin | 0 (0%) | 2 (13.3%) | 2 (4.2%) |
| Metoprolol Tartrate | 1 (3%) | 1 (6.7%) | 2 (4.2%) |
| Midazolam | 2 (6.1%) | 0 (0%) | 2 (4.2%) |
| Midazolam 50 mg/NS | 1 (3%) | 1 (6.7%) | 2 (4.2%) |
| Nelfinavir Mesylate | 2 (6.1%) | 0 (0%) | 2 (4.2%) |
| Normal Saline Flush | 1 (3%) | 1 (6.7%) | 2 (4.2%) |
| Ondasetron | 2 (6.1%) | 0 (0%) | 2 (4.2%) |
| PARENTERAL ELECTROLYTES | 2 (6.1%) | 0 (0%) | 2 (4.2%) |
| Potassium Acetate | 2 (6.1%) | 0 (0%) | 2 (4.2%) |
| Prednisone | 1 (3%) | 1 (6.7%) | 2 (4.2%) |
| Sodium Phosphate 10mm/NS | 1 (3%) | 1 (6.7%) | 2 (4.2%) |
| Thiamine 200mg/d5w | 1 (3%) | 1 (6.7%) | 2 (4.2%) |
| Thiamine 200mg/D5w | 1 (3%) | 1 (6.7%) | 2 (4.2%) |
| Trimethoprim/Sulfamethoxazole (Bactrim DS) | 1 (3%) | 1 (6.7%) | 2 (4.2%) |
| Valsartan | 1 (3%) | 1 (6.7%) | 2 (4.2%) |
| Vancomycin | 2 (6.1%) | 0 (0%) | 2 (4.2%) |
| Vit C | 2 (6.1%) | 0 (0%) | 2 (4.2%) |
| Vit D | 2 (6.1%) | 0 (0%) | 2 (4.2%) |
| Absorbic Acid | 1 (3%) | 0 (0%) | 1 (2.1%) |
| Acetaminophen (Tylenol Caplets) | 1 (3%) | 0 (0%) | 1 (2.1%) |
| Acetaminophen Liquid | 1 (3%) | 0 (0%) | 1 (2.1%) |
| Actemra (Tocilizumab) | 1 (3%) | 0 (0%) | 1 (2.1%) |
| Albumin | 1 (3%) | 0 (0%) | 1 (2.1%) |
| Albumin Human 25% | 1 (3%) | 0 (0%) | 1 (2.1%) |
| Allopurinol | 0 (0%) | 1 (6.7%) | 1 (2.1%) |
| Alprazolam | 1 (3%) | 0 (0%) | 1 (2.1%) |
| Alteplace Recombinant 6mg/NS | 1 (3%) | 0 (0%) | 1 (2.1%) |
| Alteplase Recombinant | 1 (3%) | 0 (0%) | 1 (2.1%) |
| Alteplase Recombinant 16mg/NS | 1 (3%) | 0 (0%) | 1 (2.1%) |
| Alteplase, Recombinant (Cathflo Activase) | 1 (3%) | 0 (0%) | 1 (2.1%) |
| Alteplase, Recombinant 25 mg/NS | 1 (3%) | 0 (0%) | 1 (2.1%) |
| Amino Acids/Glycerin/Electrolytes | 1 (3%) | 0 (0%) | 1 (2.1%) |
| Amiodarone | 1 (3%) | 0 (0%) | 1 (2.1%) |
| Amiodarone 900mg/D5W | 1 (3%) | 0 (0%) | 1 (2.1%) |
| Amiodaronev300mg/d5w | 1 (3%) | 0 (0%) | 1 (2.1%) |
| Amlodipine Besylate (Norvasc) | 1 (3%) | 0 (0%) | 1 (2.1%) |
| Amylodipine | 0 (0%) | 1 (6.7%) | 1 (2.1%) |
| Aptiom | 0 (0%) | 1 (6.7%) | 1 (2.1%) |
| Ascorbic Acid 1500 mg/NS | 1 (3%) | 0 (0%) | 1 (2.1%) |
| Ascorbic Acid 3000mg/Normal Saline | 1 (3%) | 0 (0%) | 1 (2.1%) |
| Ascorbid Acid in Normal Saline | 1 (3%) | 0 (0%) | 1 (2.1%) |
| Ateplase, Recombinant (Cathflo Activase) | 1 (3%) | 0 (0%) | 1 (2.1%) |
| ATORVASTATIN | 0 (0%) | 1 (6.7%) | 1 (2.1%) |
| Atorvastatin Calcium (Atorvastatin) | 1 (3%) | 0 (0%) | 1 (2.1%) |
| azithromycin | 1 (3%) | 0 (0%) | 1 (2.1%) |
| Azithromycin 250 mg/NS | 1 (3%) | 0 (0%) | 1 (2.1%) |
| Azithromycin / normal saline | 1 (3%) | 0 (0%) | 1 (2.1%) |
| Azithromycin 250/NS | 1 (3%) | 0 (0%) | 1 (2.1%) |
| Azithromycin 250mg/100ml NS | 1 (3%) | 0 (0%) | 1 (2.1%) |
| Azithromycin 500mg | 1 (3%) | 0 (0%) | 1 (2.1%) |
| Azithromycin 500mg, NS | 1 (3%) | 0 (0%) | 1 (2.1%) |
| Azithromycin/ normal saline | 1 (3%) | 0 (0%) | 1 (2.1%) |
| Bactrim DS | 1 (3%) | 0 (0%) | 1 (2.1%) |
| Bivalrudin 250mg/NS | 1 (3%) | 0 (0%) | 1 (2.1%) |
| Bupropion | 1 (3%) | 0 (0%) | 1 (2.1%) |
| Calcium Gluconate | 1 (3%) | 0 (0%) | 1 (2.1%) |
| Calcium Gluconate 10 ml/NS | 1 (3%) | 0 (0%) | 1 (2.1%) |
| Carbidopa/Levodopa (Sinemet 25/250) | 1 (3%) | 0 (0%) | 1 (2.1%) |
| Carvedilol | 1 (3%) | 0 (0%) | 1 (2.1%) |
| Ceftriaxone | 1 (3%) | 0 (0%) | 1 (2.1%) |
| Celecoxib (Celebrex%) | 1 (3%) | 0 (0%) | 1 (2.1%) |
| CHOLECALCIFEROL | 0 (0%) | 1 (6.7%) | 1 (2.1%) |
| Cholecalciferol (Vitamin D3%) | 1 (3%) | 0 (0%) | 1 (2.1%) |
| Cholecalciiferol | 1 (3%) | 0 (0%) | 1 (2.1%) |
| Cholecaliferol | 1 (3%) | 0 (0%) | 1 (2.1%) |
| Citalopram | 1 (3%) | 0 (0%) | 1 (2.1%) |
| Clonazepam | 0 (0%) | 1 (6.7%) | 1 (2.1%) |
| Clonidine Patch (Catapres-Tts-3) | 1 (3%) | 0 (0%) | 1 (2.1%) |
| Colchiine | 1 (3%) | 0 (0%) | 1 (2.1%) |
| D5w | 1 (3%) | 0 (0%) | 1 (2.1%) |
| D5W | 1 (3%) | 0 (0%) | 1 (2.1%) |
| D5W/Water | 1 (3%) | 0 (0%) | 1 (2.1%) |
| Daptomycin 500 mg/NS | 1 (3%) | 0 (0%) | 1 (2.1%) |
| DAS | 1 (3%) | 0 (0%) | 1 (2.1%) |
| DAS-181 | 1 (3%) | 0 (0%) | 1 (2.1%) |
| Dexamethasone | 0 (0%) | 1 (6.7%) | 1 (2.1%) |
| Dexmedetomidine 400 mcg/NS | 0 (0%) | 1 (6.7%) | 1 (2.1%) |
| Dextrose 5% | 1 (3%) | 0 (0%) | 1 (2.1%) |
| Dextrose 5% Water | 1 (3%) | 0 (0%) | 1 (2.1%) |
| Dextrose 50% prn | 0 (0%) | 1 (6.7%) | 1 (2.1%) |
| Dicyclomine | 0 (0%) | 1 (6.7%) | 1 (2.1%) |
| Diflucan | 1 (3%) | 0 (0%) | 1 (2.1%) |
| Dilaudid | 1 (3%) | 0 (0%) | 1 (2.1%) |
| Diphenhydramine | 1 (3%) | 0 (0%) | 1 (2.1%) |
| Diphenoxylate (Lomotil) | 1 (3%) | 0 (0%) | 1 (2.1%) |
| Diphenoxylate-atropine | 0 (0%) | 1 (6.7%) | 1 (2.1%) |
| Diphoxylate HCL/Atropine (Lomotil) | 1 (3%) | 0 (0%) | 1 (2.1%) |
| Donepezil (Aricept) | 1 (3%) | 0 (0%) | 1 (2.1%) |
| Dopamine | 1 (3%) | 0 (0%) | 1 (2.1%) |
| Doxycycline Hyclate | 1 (3%) | 0 (0%) | 1 (2.1%) |
| Doxycyline Hyclate | 1 (3%) | 0 (0%) | 1 (2.1%) |
| Doxycyline Hyclate 100mg/NS | 1 (3%) | 0 (0%) | 1 (2.1%) |
| Eloquis | 1 (3%) | 0 (0%) | 1 (2.1%) |
| Enoxaparan Sodium | 1 (3%) | 0 (0%) | 1 (2.1%) |
| Enoxaparin Sodium | 1 (3%) | 0 (0%) | 1 (2.1%) |
| Enoxaprin | 1 (3%) | 0 (0%) | 1 (2.1%) |
| Ensure high Protein | 0 (0%) | 1 (6.7%) | 1 (2.1%) |
| Enteral Formula | 1 (3%) | 0 (0%) | 1 (2.1%) |
| Enteral Nutrition | 1 (3%) | 0 (0%) | 1 (2.1%) |
| Enteral Nutrition (Glucerna%) | 1 (3%) | 0 (0%) | 1 (2.1%) |
| Epoetin Alfa | 1 (3%) | 0 (0%) | 1 (2.1%) |
| Ertapenem Sodium 1 gm | 0 (0%) | 1 (6.7%) | 1 (2.1%) |
| Esmolol (Brevibloc) | 1 (3%) | 0 (0%) | 1 (2.1%) |
| Esmolol HCL 2000mg/NS | 1 (3%) | 0 (0%) | 1 (2.1%) |
| Etomidate | 0 (0%) | 1 (6.7%) | 1 (2.1%) |
| FAMOTIDINE | 1 (3%) | 0 (0%) | 1 (2.1%) |
| Famotidine (Pepcid I.v.) | 1 (3%) | 0 (0%) | 1 (2.1%) |
| Fentanyl | 1 (3%) | 0 (0%) | 1 (2.1%) |
| Fentanyl Citrate 25mg | 1 (3%) | 0 (0%) | 1 (2.1%) |
| Fentynal Citrate | 1 (3%) | 0 (0%) | 1 (2.1%) |
| Filgrastim | 1 (3%) | 0 (0%) | 1 (2.1%) |
| Finasteride (Proscar) | 1 (3%) | 0 (0%) | 1 (2.1%) |
| Fluconazole (Diflucan) | 1 (3%) | 0 (0%) | 1 (2.1%) |
| Fluconazole/NS | 1 (3%) | 0 (0%) | 1 (2.1%) |
| Fluconizole | 1 (3%) | 0 (0%) | 1 (2.1%) |
| Fluticasone | 1 (3%) | 0 (0%) | 1 (2.1%) |
| Fluticasone Proprionate (Flonase) 0.05% | 1 (3%) | 0 (0%) | 1 (2.1%) |
| Folic Acid | 1 (3%) | 0 (0%) | 1 (2.1%) |
| Furosemide 100 mg/NS | 1 (3%) | 0 (0%) | 1 (2.1%) |
| Furosemide 100mg/NS | 1 (3%) | 0 (0%) | 1 (2.1%) |
| Gabapentin | 1 (3%) | 0 (0%) | 1 (2.1%) |
| Gabapentin (Neurontin) | 1 (3%) | 0 (0%) | 1 (2.1%) |
| Gemfibrozil | 1 (3%) | 0 (0%) | 1 (2.1%) |
| Gemfibrozil (Lopid) | 1 (3%) | 0 (0%) | 1 (2.1%) |
| Guaifenesin | 0 (0%) | 1 (6.7%) | 1 (2.1%) |
| Haloperidol | 1 (3%) | 0 (0%) | 1 (2.1%) |
| Heparin | 1 (3%) | 0 (0%) | 1 (2.1%) |
| Heparin Sodium (Porcine) | 1 (3%) | 0 (0%) | 1 (2.1%) |
| Human insulin Reguar | 1 (3%) | 0 (0%) | 1 (2.1%) |
| HYDROXYCHLOROQUINE SULFATE | 1 (3%) | 0 (0%) | 1 (2.1%) |
| Hydroxychloroquine Sulfate (Plaquenil) | 0 (0%) | 1 (6.7%) | 1 (2.1%) |
| Hydroxychlroquine | 1 (3%) | 0 (0%) | 1 (2.1%) |
| Hydroxyzine | 0 (0%) | 1 (6.7%) | 1 (2.1%) |
| hyrdroxychloroquine | 1 (3%) | 0 (0%) | 1 (2.1%) |
| Hyrdroxychloroquine | 0 (0%) | 1 (6.7%) | 1 (2.1%) |
| Ibuprofen | 0 (0%) | 1 (6.7%) | 1 (2.1%) |
| Insulin Detemir | 0 (0%) | 1 (6.7%) | 1 (2.1%) |
| Insulin Human Regular 100 units/NS | 1 (3%) | 0 (0%) | 1 (2.1%) |
| Insulin Regular Human | 0 (0%) | 1 (6.7%) | 1 (2.1%) |
| Intraconazole | 1 (3%) | 0 (0%) | 1 (2.1%) |
| Irbesartan (Avapro) | 0 (0%) | 1 (6.7%) | 1 (2.1%) |
| Isosorbide mono nitrate | 1 (3%) | 0 (0%) | 1 (2.1%) |
| Iveremectin | 1 (3%) | 0 (0%) | 1 (2.1%) |
| IVERMECTIN | 1 (3%) | 0 (0%) | 1 (2.1%) |
| Kcl | 1 (3%) | 0 (0%) | 1 (2.1%) |
| KCL | 1 (3%) | 0 (0%) | 1 (2.1%) |
| KCL 40mEq/ NS | 0 (0%) | 1 (6.7%) | 1 (2.1%) |
| Lactobacillus Acidoph/Bulgaricus | 1 (3%) | 0 (0%) | 1 (2.1%) |
| Lactulose (Lactulose 20 Gm/30ml) | 1 (3%) | 0 (0%) | 1 (2.1%) |
| Lactulose 20 gm/30ml | 1 (3%) | 0 (0%) | 1 (2.1%) |
| Lasix | 1 (3%) | 0 (0%) | 1 (2.1%) |
| Latanoprost (Xalatan) | 1 (3%) | 0 (0%) | 1 (2.1%) |
| Levophed | 1 (3%) | 0 (0%) | 1 (2.1%) |
| LevophedDrip | 1 (3%) | 0 (0%) | 1 (2.1%) |
| LEVOTHYROXINE | 1 (3%) | 0 (0%) | 1 (2.1%) |
| Linezolid | 1 (3%) | 0 (0%) | 1 (2.1%) |
| Liposyn III 20% | 1 (3%) | 0 (0%) | 1 (2.1%) |
| Lisinopril (Zestril) | 0 (0%) | 1 (6.7%) | 1 (2.1%) |
| Loperamide | 1 (3%) | 0 (0%) | 1 (2.1%) |
| Loratadine | 1 (3%) | 0 (0%) | 1 (2.1%) |
| LOVENOX | 1 (3%) | 0 (0%) | 1 (2.1%) |
| Mag sulfate | 1 (3%) | 0 (0%) | 1 (2.1%) |
| Magnesium Sulfate 2 gm/Sodium Chloride | 1 (3%) | 0 (0%) | 1 (2.1%) |
| magnesium sulfate 2gm/NS | 1 (3%) | 0 (0%) | 1 (2.1%) |
| Magnesium Sulfate 2gm/NS | 0 (0%) | 1 (6.7%) | 1 (2.1%) |
| Magnesium Sulfate 2gm/Sodium Chloride | 1 (3%) | 0 (0%) | 1 (2.1%) |
| Magnesium Sulfate 3 gm/NS | 1 (3%) | 0 (0%) | 1 (2.1%) |
| Magnesium Sulfate/D5W | 1 (3%) | 0 (0%) | 1 (2.1%) |
| Medroxyprogesterone | 0 (0%) | 1 (6.7%) | 1 (2.1%) |
| MELATONIN | 1 (3%) | 0 (0%) | 1 (2.1%) |
| Melatonin (Melatonin) | 1 (3%) | 0 (0%) | 1 (2.1%) |
| Memantine (Namenda) | 1 (3%) | 0 (0%) | 1 (2.1%) |
| Mereponem | 1 (3%) | 0 (0%) | 1 (2.1%) |
| Meropenem 1 gm | 1 (3%) | 0 (0%) | 1 (2.1%) |
| Meropenem 1 gm/NS | 1 (3%) | 0 (0%) | 1 (2.1%) |
| Meropenem 500 mg/Sodium Chloride | 1 (3%) | 0 (0%) | 1 (2.1%) |
| Meropenem 500mg/NS | 0 (0%) | 1 (6.7%) | 1 (2.1%) |
| Meropenem 750 mg/NS | 1 (3%) | 0 (0%) | 1 (2.1%) |
| Meropenem 750mg/NS | 1 (3%) | 0 (0%) | 1 (2.1%) |
| Metaclopramide HCL (Reglan) | 1 (3%) | 0 (0%) | 1 (2.1%) |
| Methlyprednisolone | 1 (3%) | 0 (0%) | 1 (2.1%) |
| Methlyprednisolone Sodium Succinate | 1 (3%) | 0 (0%) | 1 (2.1%) |
| Methylpredisolone | 1 (3%) | 0 (0%) | 1 (2.1%) |
| methylprednisolone Sodium Succinate | 1 (3%) | 0 (0%) | 1 (2.1%) |
| Methylprednisolone Sodium Succinate 1000mg/NS | 0 (0%) | 1 (6.7%) | 1 (2.1%) |
| Methylprednisolone1000 mg/ Normal Saline | 1 (3%) | 0 (0%) | 1 (2.1%) |
| Methyprednisolone | 1 (3%) | 0 (0%) | 1 (2.1%) |
| Metronidazole | 0 (0%) | 1 (6.7%) | 1 (2.1%) |
| Midazolam (Versed) | 1 (3%) | 0 (0%) | 1 (2.1%) |
| Midazolam 50mg/NS | 1 (3%) | 0 (0%) | 1 (2.1%) |
| Midazolam Drip | 1 (3%) | 0 (0%) | 1 (2.1%) |
| Mineral Oil/White Petrolatum | 0 (0%) | 1 (6.7%) | 1 (2.1%) |
| Minoxidil | 1 (3%) | 0 (0%) | 1 (2.1%) |
| Montelukast Sodium | 1 (3%) | 0 (0%) | 1 (2.1%) |
| Morphine sulfate | 0 (0%) | 1 (6.7%) | 1 (2.1%) |
| MORPHINE SULFATE | 1 (3%) | 0 (0%) | 1 (2.1%) |
| Multi Vitamin w/ Chromium/Copper, Manganese, Insulin Human R 30 U/Amino Acids, Calcium | 1 (3%) | 0 (0%) | 1 (2.1%) |
| Multivitamin | 1 (3%) | 0 (0%) | 1 (2.1%) |
| Mycophenolate | 1 (3%) | 0 (0%) | 1 (2.1%) |
| MYCOPHENOLATE MOFETIL | 1 (3%) | 0 (0%) | 1 (2.1%) |
| NELFINAVIR | 1 (3%) | 0 (0%) | 1 (2.1%) |
| Nelfinavir 1250mg | 1 (3%) | 0 (0%) | 1 (2.1%) |
| Nicardipine 25mg/NS | 1 (3%) | 0 (0%) | 1 (2.1%) |
| Nicotine (Nicoderm Cq 21 Mg/24 Hr Patch) | 1 (3%) | 0 (0%) | 1 (2.1%) |
| Nicotine 21 mg/24 hr patch | 1 (3%) | 0 (0%) | 1 (2.1%) |
| Norepinephrine Bitartrate 4 mg/D5W | 0 (0%) | 1 (6.7%) | 1 (2.1%) |
| Norepinephrine Bitartrate 4mg/Dextrose | 1 (3%) | 0 (0%) | 1 (2.1%) |
| Normal Saline | 1 (3%) | 0 (0%) | 1 (2.1%) |
| Normal Saline Lock Flush | 1 (3%) | 0 (0%) | 1 (2.1%) |
| NS 3 ml Flush | 0 (0%) | 1 (6.7%) | 1 (2.1%) |
| NS Lock Flush | 1 (3%) | 0 (0%) | 1 (2.1%) |
| Nystatin | 0 (0%) | 1 (6.7%) | 1 (2.1%) |
| Ocean Nasal Spray /NS | 1 (3%) | 0 (0%) | 1 (2.1%) |
| pantoprazole sodium (Protonix) | 1 (3%) | 0 (0%) | 1 (2.1%) |
| PAROXETINE | 1 (3%) | 0 (0%) | 1 (2.1%) |
| Paroxetine (Paxil) | 1 (3%) | 0 (0%) | 1 (2.1%) |
| Phenergan | 1 (3%) | 0 (0%) | 1 (2.1%) |
| Phenyleprhine 25 mg/NS | 1 (3%) | 0 (0%) | 1 (2.1%) |
| Plasma Lyte Injection | 1 (3%) | 0 (0%) | 1 (2.1%) |
| Plasma-Lyte Injection | 1 (3%) | 0 (0%) | 1 (2.1%) |
| Polyethylene Glycol (Clearlax) | 1 (3%) | 0 (0%) | 1 (2.1%) |
| Potassium Acetate 40 meQ | 1 (3%) | 0 (0%) | 1 (2.1%) |
| Potassium Acetate 40 mEq/NS | 0 (0%) | 1 (6.7%) | 1 (2.1%) |
| Potassium Acetate 40 meq/Sodium Chloride | 1 (3%) | 0 (0%) | 1 (2.1%) |
| Potassium Bicarbonate | 0 (0%) | 1 (6.7%) | 1 (2.1%) |
| Potassium Bicarbonate/Citric Acid | 1 (3%) | 0 (0%) | 1 (2.1%) |
| Potassium Bicarbonate/Citric Acid (Effer-K) | 1 (3%) | 0 (0%) | 1 (2.1%) |
| potassium Chloride | 1 (3%) | 0 (0%) | 1 (2.1%) |
| Potassium Chloride 40 meq | 1 (3%) | 0 (0%) | 1 (2.1%) |
| Potassium Chloride 40 mEq | 1 (3%) | 0 (0%) | 1 (2.1%) |
| Potassium Chloride 40 mEq/NS | 1 (3%) | 0 (0%) | 1 (2.1%) |
| Potassium Phos/Sodium Phos (Neutra-Phos Packet) | 1 (3%) | 0 (0%) | 1 (2.1%) |
| Potassium Phos/Sodium Phos (Neutra-Phos Pcket | 1 (3%) | 0 (0%) | 1 (2.1%) |
| Potassium Phosphate (Neutra-Phos-K Packet) | 1 (3%) | 0 (0%) | 1 (2.1%) |
| PREDNISONE | 1 (3%) | 0 (0%) | 1 (2.1%) |
| Promethazine HCl (Phenergan) | 1 (3%) | 0 (0%) | 1 (2.1%) |
| Propofol | 0 (0%) | 1 (6.7%) | 1 (2.1%) |
| Pt denies any medications | 0 (0%) | 1 (6.7%) | 1 (2.1%) |
| Queetiapine Fumarate (SerOQUEL) | 1 (3%) | 0 (0%) | 1 (2.1%) |
| Questran Light | 1 (3%) | 0 (0%) | 1 (2.1%) |
| Remdesivir 100 mg/NS | 1 (3%) | 0 (0%) | 1 (2.1%) |
| Remdesivir/Normal Saline | 1 (3%) | 0 (0%) | 1 (2.1%) |
| Rivaroxaban (Xarelto) | 1 (3%) | 0 (0%) | 1 (2.1%) |
| Rocuronium | 1 (3%) | 0 (0%) | 1 (2.1%) |
| Rocuronium Bromide | 0 (0%) | 1 (6.7%) | 1 (2.1%) |
| Rocuronium Bromide (Zemuron) | 1 (3%) | 0 (0%) | 1 (2.1%) |
| Sertraline | 0 (0%) | 1 (6.7%) | 1 (2.1%) |
| Sertraline HCl (Zoloft) | 1 (3%) | 0 (0%) | 1 (2.1%) |
| Sildenafil Citrate | 1 (3%) | 0 (0%) | 1 (2.1%) |
| Sodium Bicarbonate 150 ml/D5W/Water | 1 (3%) | 0 (0%) | 1 (2.1%) |
| Sodium Bicarbonate 8.4% | 1 (3%) | 0 (0%) | 1 (2.1%) |
| Sodium Chloride (Salinex) | 1 (3%) | 0 (0%) | 1 (2.1%) |
| Sodium Chloride 0.9%/Pitressin Synthetic | 1 (3%) | 0 (0%) | 1 (2.1%) |
| Sodium Hypochlorite (Dakin's solution half strength) | 1 (3%) | 0 (0%) | 1 (2.1%) |
| Sodium Hypochlorite (Dakin's Solution) | 1 (3%) | 0 (0%) | 1 (2.1%) |
| Sodium Phosphate | 1 (3%) | 0 (0%) | 1 (2.1%) |
| Sodium Phosphate 10mm/Sodium Chloride | 1 (3%) | 0 (0%) | 1 (2.1%) |
| Sodium Phosphate 20 mm/NS | 0 (0%) | 1 (6.7%) | 1 (2.1%) |
| Sterile Water | 1 (3%) | 0 (0%) | 1 (2.1%) |
| Stool Softener/ Laxative of Choice | 1 (3%) | 0 (0%) | 1 (2.1%) |
| Tamsulosin | 1 (3%) | 0 (0%) | 1 (2.1%) |
| Tamsulosin HCl (Flomax) | 1 (3%) | 0 (0%) | 1 (2.1%) |
| Testosterone | 1 (3%) | 0 (0%) | 1 (2.1%) |
| Testosterone Cypionate | 0 (0%) | 1 (6.7%) | 1 (2.1%) |
| Theragran (multi vitamin) | 1 (3%) | 0 (0%) | 1 (2.1%) |
| Thiamine | 1 (3%) | 0 (0%) | 1 (2.1%) |
| Thiamine 200 mg/D5W | 1 (3%) | 0 (0%) | 1 (2.1%) |
| Thiamine 200/d5w | 1 (3%) | 0 (0%) | 1 (2.1%) |
| Thiamine 200mg/d5W | 1 (3%) | 0 (0%) | 1 (2.1%) |
| Thiamine 200mg/Dextrose | 0 (0%) | 1 (6.7%) | 1 (2.1%) |
| Thiamine HCI 200 mg/Dextrose | 1 (3%) | 0 (0%) | 1 (2.1%) |
| Thiamine HCl 200 mg/Dextrose | 1 (3%) | 0 (0%) | 1 (2.1%) |
| Thiamine HCL/Dextrose | 1 (3%) | 0 (0%) | 1 (2.1%) |
| Thiamine/D5W | 1 (3%) | 0 (0%) | 1 (2.1%) |
| Tocilizumab (Actembra) | 0 (0%) | 1 (6.7%) | 1 (2.1%) |
| Tocilizumab (Actemra) | 1 (3%) | 0 (0%) | 1 (2.1%) |
| Total Parenteral Nutrition with Lipids | 0 (0%) | 1 (6.7%) | 1 (2.1%) |
| TPA Infusion | 1 (3%) | 0 (0%) | 1 (2.1%) |
| Tramadol HCl (Ultram) | 1 (3%) | 0 (0%) | 1 (2.1%) |
| Triamcinolone Lotion | 1 (3%) | 0 (0%) | 1 (2.1%) |
| Triamethoprim/Sulfamethoxazole DS | 1 (3%) | 0 (0%) | 1 (2.1%) |
| trimethoprim/suflamethoxazole (Bactrim DS) | 1 (3%) | 0 (0%) | 1 (2.1%) |
| Trimethoprim/Sulfamethoxasole | 1 (3%) | 0 (0%) | 1 (2.1%) |
| Trimethoprim/Sulfamethoxasole DS | 1 (3%) | 0 (0%) | 1 (2.1%) |
| trimethoprim/sulfamethoxazone DS | 1 (3%) | 0 (0%) | 1 (2.1%) |
| TYLENOL | 0 (0%) | 1 (6.7%) | 1 (2.1%) |
| Tylenol #3 | 1 (3%) | 0 (0%) | 1 (2.1%) |
| Tylenol 325mg | 1 (3%) | 0 (0%) | 1 (2.1%) |
| Valsarten | 0 (0%) | 1 (6.7%) | 1 (2.1%) |
| Vancomycin 1000 mg/NS | 1 (3%) | 0 (0%) | 1 (2.1%) |
| Vancomycin 1250 mg/NS | 1 (3%) | 0 (0%) | 1 (2.1%) |
| Vancomycin HCl 1000 mg/NS | 1 (3%) | 0 (0%) | 1 (2.1%) |
| Vancomycin HCl 1000 mg/Sodiumm Chloride | 1 (3%) | 0 (0%) | 1 (2.1%) |
| Vasopressin Drip | 1 (3%) | 0 (0%) | 1 (2.1%) |
| Vitamin A/ Vitamin D Ointment | 1 (3%) | 0 (0%) | 1 (2.1%) |
| Vitamin B1 (Thiamine) | 1 (3%) | 0 (0%) | 1 (2.1%) |
| Vitamin C | 1 (3%) | 0 (0%) | 1 (2.1%) |
| Warfarin | 1 (3%) | 0 (0%) | 1 (2.1%) |
| zinc sulfate | 1 (3%) | 0 (0%) | 1 (2.1%) |
| Zofran | 1 (3%) | 0 (0%) | 1 (2.1%) |
| Zolipidem | 1 (3%) | 0 (0%) | 1 (2.1%) |
| Zolipidem Tartrate | 1 (3%) | 0 (0%) | 1 (2.1%) |
| Zyvox | 1 (3%) | 0 (0%) | 1 (2.1%) |
|  |  |  |  |

Supplementary Table 2. Full table of adverse events.

| **Characteristic** | **HB-adMSCs 100MM**, N = 33 | | **Placebo**, N = 15 | | **Overall**, N = 48 | |
| --- | --- | --- | --- | --- | --- | --- |
|  | **N (%)** | **Events** | **N (%)** | **Events** | **N (%)** | **Events** |
| **Number of Subjects** | 33 | 80 | 15 | 39 | 48 | 119 |
| **AE/SAE, n (%)** |  |  |  |  |  |  |
| Body Aches | 7 (21.2%) | 9 | 4 (26.7%) | 5 | 11 (22.9%) | 14 |
| Dyspnea | 7 (21.2%) | 7 | 2 (13.3%) | 2 | 9 (18.8%) | 9 |
| Cardiopulmonary Failure | 6 (18.2%) | 6 | 2 (13.3%) | 2 | 8 (16.7%) | 8 |
| Cough | 5 (15.2%) | 5 | 3 (20.0%) | 3 | 8 (16.7%) | 8 |
| Headache | 2 (6.1%) | 4 | 3 (20.0%) | 4 | 5 (10.4%) | 4 |
| Hyperthermia | 4 (12.1%) | 6 | 2 (13.3%) | 2 | 6 (12.5%) | 8 |
| Fatigue | 2 (6.1%) | 2 | 4 (26.7%) | 4 | 6 (12.5%) | 6 |
| Anxiety | 4 (12.1%) | 4 | 0 (0.0%) |  | 4 (8.3%) | 4 |
| Hypertension | 2 (6.1%) | 2 | 2 (13.3%) | 2 | 4 (8.3%) | 4 |
| Anemia | 3 (9.1%) | 3 | 0 (0.0%) |  | 3 (6.2%) | 3 |
| Chest Discomfort | 1 (3.0%) | 1 | 2 (13.3%) | 2 | 3 (6.2%) | 3 |
| Chills | 1 (3.0%) | 1 | 1 (6.7%) | 2 | 2 (4.2%) | 3 |
| Diarrhea | 3 (9.1%) | 3 | 0 (0.0%) |  | 3 (6.2%) | 3 |
| Rash, Vesicular | 1 (3.0%) | 1 | 0 (0.0%) |  | 1 (2.1%) | 1 |
| Agitation | 0 (0.0%) |  | 2 (13.3%) | 2 | 2 (4.2%) | 2 |
| Bradycardia | 1 (3.0%) | 1 | 0 (0.0%) |  | 1 (2.1%) | 1 |
| Nausea | 0 (0.0%) |  | 1 (6.7%) | 1 | 1 (2.1%) | 1 |
| Painful Respiration | 1 (3.0%) | 1 | 1 (6.7%) | 1 | 2 (4.2%) | 2 |
| Constipation | 0 (0.0%) |  | 1 (6.7%) | 1 | 1 (2.1%) | 1 |
| Pancreatitis | 0 (0.0%) |  | 1 (6.7%) | 1 | 1 (2.1%) | 1 |
| Poor Venous Access | 1 (3.0%) | 1 | 1 (6.7%) | 1 | 2 (4.2%) | 2 |
| ESR Raised | 1 (3.0%) | 1 | 0 (0.0%) |  | 1 (2.1%) | 1 |
| Myofascial Neck Pain | 1 (3.0%) | 1 | 0 (0.0%) |  | 1 (2.1%) | 1 |
| Sepsis | 1 (3.0%) | 1 | 0 (0.0%) |  | 1 (2.1%) | 1 |
| Brain Natriuretic Peptide Increased | 1 (3.0%) | 1 | 0 (0.0%) |  | 1 (2.1%) | 1 |
| Carbon Dioxide Abnormal | 1 (3.0%) | 1 | 0 (0.0%) |  | 1 (2.1%) | 1 |
| Hyperglycemia | 1 (3.0%) | 1 | 0 (0.0%) |  | 1 (2.1%) | 1 |
| Urine Abnormality | 1 (3.0%) | 1 | 0 (0.0%) |  | 1 (2.1%) | 1 |
| Pleural Effusion | 1 (3.0%) | 1 | 0 (0.0%) |  | 1 (2.1%) | 1 |
| Myocardial Necrosis Marker Increased | 1 (3.0%) | 1 | 0 (0.0%) |  | 1 (2.1%) | 1 |
| Blood Electrolytes, Abnormal | 1 (3.0%) | 1 | 0 (0.0%) |  | 1 (2.1%) | 1 |
| Coagulopathy | 2 (6.1%) | 2 | 0 (0.0%) |  | 2 (4.2%) | 2 |
| WBC, Elevated | 1 (3.0%) | 1 | 0 (0.0%) |  | 1 (2.1%) | 1 |
| Respiratory Failure | 1 (3.0%) | 1 | 0 (0.0%) |  | 1 (2.1%) | 1 |
| Dry Throat | 0 (0.0%) |  | 1 (6.7%) | 1 | 1 (2.1%) | 1 |
| Confusional State | 2 (6.1%) | 2 | 0 (0.0%) | 2 | 2 (4.2%) | 4 |
| Decreased Appetite | 1 (3.0%) | 1 | 0 (0.0%) |  | 1 (2.1%) | 1 |
| Dysphagia | 1 (3.0%) | 1 | 0 (0.0%) |  | 1 (2.1%) | 1 |
| Feeling Abnormal | 0 (0.0%) |  | 1 (6.7%) | 1 | 1 (2.1%) | 1 |
| Fall | 0 (0.0%) |  | 1 (6.7%) | 1 | 1 (2.1%) | 1 |
| Depression | 0 (0.0%) |  | 1 (6.7%) | 1 | 1 (2.1%) | 1 |
| Arrythmias, Cardiac | 1 (3.0%) | 1 | 0 (0.0%) |  | 1 (2.1%) | 1 |
| Kidney Failure | 1 (3.0%) | 1 | 0 (0.0%) |  | 1 (2.1%) | 1 |
| Hypotension | 1 (3.0%) | 1 | 0 (0.0%) |  | 1 (2.1%) | 1 |
| Puncture Site Bleeding | 1 (3.0%) | 1 | 0 (0.0%) |  | 1 (2.1%) | 1 |
| Joint Pain | 1 (3.0%) | 1 | 0 (0.0%) |  | 1 (2.1%) | 1 |
|  |  |  |  |  |  |  |
| **Adverse Event Severity, n (%)** |  |  |  |  |  |  |
| Mild | 25 (75.8%) | 68 | 12 (80.0%) | 37 | 37 (77.1%) | 105 |
| Moderate | 2 (6.1%) | 2 | 0 (0.0%) |  | 2 (4.2%) | 2 |
| Severe | 1 (3.0%) | 1 | 0 (0.0%) |  | 1 (2.1%) | 1 |
| Life-threatening | 1 (3.0%) | 1 | 0 (0.0%) |  | 1 (2.1%) | 1 |
| Fatal | 8 (24.2%) | 8 | 2 (13.3%) | 2 | 10 (20.8%) | 10 |
|  |  |  |  |  |  |  |
| **Attribution to Study Drug, n (%)** |  |  |  |  |  |  |
| Definite |  |  |  |  |  |  |
| Probable |  |  |  |  |  |  |
| Possible |  |  |  |  |  |  |
| Unlikely | 8 (24.2%) | 18 | 8 (53.3%) | 15 | 16 (33.3%) | 33 |
| Unrelated | 24 (72.7%) | 62 | 11 (73.3%) | 24 | 35 (72.9%) | 86 |
| **Serious, n (%)** | 9 (26.5%) | 10 | 2 (14.3%) | 2 | 11 (22.9%) | 12 |
| **Action, n (%)** |  |  |  |  |  |  |
| None | 27 (81.8%) | 80 | 11 (73.3%) | 38 | 38 (79.2%) | 118 |
| Interrupted |  |  |  |  |  |  |
| Discontinued |  |  | 1 (6.7%) | 1 |  |  |
| Dose reduced |  |  |  |  |  |  |
| Dose increased |  |  |  |  |  |  |
|  |  |  |  |  |  |  |
| **Outcome, n (%)** |  |  |  |  |  |  |
| Fatal | 8 (24.2%) | 8 | 2 (14.3%) | 2 | 10 (21.3%) | 10 |
| Not recovered/Not resolved |  |  |  |  |  |  |
| Recovered with Sequelae |  |  |  |  |  |  |
| Recovered without Sequelae | 25 (75.8%) | 72 | 12 (85.7%) | 37 | 37 (78.7%) | 109 |
| Recovering/Resolving |  |  |  |  |  |  |

Supplementary Table 3. Vital signs.

|  | **Infusion 1, N = 45** | | | **Infusion 2, N = 37** | | | **Infusion 3, N = 31** | | | **Infusion 4, N = 27** | | |
| --- | --- | --- | --- | --- | --- | --- | --- | --- | --- | --- | --- | --- |
| **Characteristic** | **N** | **HB-adMSC 100MM, N = 31** | **Placebo, N = 14** | **N** | **HB-adMSC 100MM, N = 25** | **Placebo, N = 12** | **N** | **HB-adMSC 100MM, N = 21** | **Placebo, N = 10** | **N** | **HB-adMSC 100MM, N = 18** | **Placebo, N = 9** |
| Weight (lbs), M (SD) | 45 | 197.1 (49.2) | 189.5 (39.7) | 37 | 197.1 (54.0) | 188.2 (44.7) | 31 | 195.8 (55.9) | 191.6 (43.4) | 27 | 206.2 (57.7) | 206.1 (61.5) |
| Respiration Rate (breaths/minute), M (SD) | 45 | 22.5 (9.7) | 22.1 (4.8) | 37 | 22.2 (6.8) | 19.3 (5.9) | 31 | 20.0 (4.2) | 19.6 (3.0) | 27 | 23.4 (16.5) | 18.4 (3.0) |
| Temperature (F), M (SD) | 45 | 98.0 (0.7) | 98.1 (0.8) | 37 | 97.7 (0.7) | 97.8 (0.6) | 31 | 97.7 (0.6) | 97.8 (0.7) | 26 | 97.9 (0.6) | 97.9 (0.4) |
| BP systolic (mmHg), M (SD) | 45 | 134.9 (19.3) | 132.9 (18.1) | 37 | 131.1 (20.4) | 128.7 (24.8) | 31 | 129.3 (18.3) | 127.4 (19.8) | 27 | 127.7 (13.8) | 138.8 (21.7) |
| BP diastolic (mmHg), M (SD) | 45 | 77.3 (12.5) | 81.0 (9.6) | 37 | 76.4 (12.4) | 74.9 (6.7) | 31 | 74.2 (13.2) | 76.3 (9.7) | 27 | 73.3 (10.0) | 81.3 (6.9) |
| Heart rate (beats/minute), M (SD) | 45 | 81.1 (14.7) | 79.9 (10.6) | 37 | 79.6 (13.4) | 80.6 (10.4) | 31 | 74.6 (16.4) | 85.0 (15.5) | 27 | 82.8 (11.3) | 84.0 (12.9) |

Supplementary Table 4. Physical examination.

|  | **Infusion 1, N = 47** | | | **Infusion 2, N = 38** | | | **Infusion 3, N = 32** | | | **Infusion 4, N = 28** | | |
| --- | --- | --- | --- | --- | --- | --- | --- | --- | --- | --- | --- | --- |
| **Characteristic** | **N** | **HB-adMSC 100MM, N = 33** | **Placebo, N = 14** | **N** | **HB-adMSC 100MM, N = 25** | **Placebo, N = 13** | **N** | **HB-adMSC 100MM, N = 22** | **Placebo, N = 10** | **N** | **HB-adMSC 100MM, N = 19** | **Placebo, N = 9** |
| HEENT, n (%) | 46 |  |  | 38 |  |  | 31 |  |  | 28 |  |  |
| Normal |  | 31 (96.9%) | 14 (100.0%) |  | 24 (96.0%) | 13 (100.0%) |  | 21 (100.0%) | 10 (100.0%) |  | 19 (100.0%) | 9 (100.0%) |
| Abnormal |  | 1 (3.1%) | 0 (0.0%) |  | 1 (4.0%) | 0 (0.0%) |  |  |  |  |  |  |
| Skin, n (%) | 46 |  |  | 38 |  |  | 31 |  |  | 28 |  |  |
| Normal |  | 32 (100.0%) | 14 (100.0%) |  | 25 (100.0%) | 13 (100.0%) |  | 21 (100.0%) | 10 (100.0%) |  | 19 (100.0%) | 9 (100.0%) |
| Cardiovascular, n (%) | 46 |  |  | 38 |  |  | 31 |  |  | 28 |  |  |
| Abnormal |  | 1 (3.1%) | 0 (0.0%) |  | 1 (4.0%) | 0 (0.0%) |  | 1 (4.8%) | 0 (0.0%) |  | 2 (10.5%) | 0 (0.0%) |
| Normal |  | 31 (96.9%) | 14 (100.0%) |  | 24 (96.0%) | 13 (100.0%) |  | 20 (95.2%) | 10 (100.0%) |  | 17 (89.5%) | 9 (100.0%) |
| Respiratory, n (%) | 46 |  |  | 38 |  |  | 31 |  |  | 28 |  |  |
| Abnormal |  | 28 (87.5%) | 13 (92.9%) |  | 24 (96.0%) | 13 (100.0%) |  | 19 (90.5%) | 9 (90.0%) |  | 16 (84.2%) | 8 (88.9%) |
| Normal |  | 3 (9.4%) | 1 (7.1%) |  | 1 (4.0%) | 0 (0.0%) |  | 2 (9.5%) | 1 (10.0%) |  | 3 (15.8%) | 1 (11.1%) |
| Not examined |  | 1 (3.1%) | 0 (0.0%) |  |  |  |  |  |  |  |  |  |
| Abdomen, n (%) | 46 |  |  | 38 |  |  | 31 |  |  | 28 |  |  |
| Abnormal |  | 0 (0.0%) | 1 (7.1%) |  | 0 (0.0%) | 1 (7.7%) |  | 1 (4.8%) | 0 (0.0%) |  | 2 (10.5%) | 0 (0.0%) |
| Normal |  | 32 (100.0%) | 13 (92.9%) |  | 25 (100.0%) | 12 (92.3%) |  | 20 (95.2%) | 10 (100.0%) |  | 17 (89.5%) | 9 (100.0%) |
| Lymph nodes, n (%) | 46 |  |  | 38 |  |  | 31 |  |  | 28 |  |  |
| Normal |  | 32 (100.0%) | 14 (100.0%) |  | 25 (100.0%) | 13 (100.0%) |  | 21 (100.0%) | 10 (100.0%) |  | 19 (100.0%) | 9 (100.0%) |
| Musculoskeletal, n (%) | 46 |  |  | 38 |  |  | 31 |  |  | 28 |  |  |
| Abnormal |  | 2 (6.2%) | 0 (0.0%) |  | 2 (8.0%) | 0 (0.0%) |  | 3 (14.3%) | 5 (50.0%) |  | 4 (21.1%) | 1 (11.1%) |
| Normal |  | 29 (90.6%) | 14 (100.0%) |  | 23 (92.0%) | 13 (100.0%) |  | 18 (85.7%) | 5 (50.0%) |  | 15 (78.9%) | 8 (88.9%) |
| Not examined |  | 1 (3.1%) | 0 (0.0%) |  |  |  |  |  |  |  |  |  |
| Neurological, n (%) | 46 |  |  | 38 |  |  | 31 |  |  | 28 |  |  |
| Abnormal |  | 2 (6.2%) | 0 (0.0%) |  | 2 (8.0%) | 1 (7.7%) |  | 4 (19.0%) | 2 (20.0%) |  | 3 (15.8%) | 1 (11.1%) |
| Normal |  | 30 (93.8%) | 14 (100.0%) |  | 23 (92.0%) | 12 (92.3%) |  | 17 (81.0%) | 8 (80.0%) |  | 16 (84.2%) | 8 (88.9%) |

Supplementary Table 5. Biochemistry parameters.

|  | **Infusion 1, N = 43** | | | **Infusion 2, N = 35** | | | **Infusion 3, N = 27** | | | **Infusion 4, N = 26** | | |
| --- | --- | --- | --- | --- | --- | --- | --- | --- | --- | --- | --- | --- |
| **Characteristic** | **N** | **HB-adMSC 100MM, N = 30** | **Placebo, N = 13** | **N** | **HB-adMSC 100MM, N = 24** | **Placebo, N = 11** | **N** | **HB-adMSC 100MM, N = 18** | **Placebo, N = 9** | **N** | **HB-adMSC 100MM, N = 17** | **Placebo, N = 9** |
| **Comprehensive Metabolic Panel** | | | | | | | | | | | | |
| Glucose (mg/dL), M (SD) | 38 | 153.4 (89.0) | 165.4 (52.5) | 31 | 148.2 (74.8) | 145.6 (58.7) | 25 | 175.7 (106.4) | 165.7 (37.5) | 24 | 166.9 (110.0) | 129.8 (74.4) |
| Blood Urea Nitrogen (mg/dL), M (SD) | 38 | 22.9 (17.4) | 15.6 (4.8) | 31 | 24.0 (18.5) | 12.7 (4.4) | 25 | 28.4 (28.1) | 19.9 (7.8) | 24 | 19.8 (12.1) | 14.9 (6.0) |
| Creatinine (mg/dL), M (SD) | 38 | 1.0 (1.2) | 0.7 (0.2) | 31 | 0.9 (0.7) | 0.7 (0.2) | 25 | 1.4 (1.8) | 0.8 (0.1) | 24 | 1.2 (1.2) | 0.8 (0.1) |
| Estimated glomerular filtration rate if Non-African American (mL/min/1.73), M (SD) | 26 | 143.0 (103.8) | 117.0 (33.9) | 23 | 113.4 (25.2) | 112.8 (22.4) | 19 | 102.8 (44.8) | 112.5 (15.9) | 16 | 107.0 (36.0) | 108.0 (18.0) |
| Estimated glomerular filtration rate If African American (mL/min/1.73), M (SD) | 8 | 74.3 (42.7) | 107.0 (NA) | 5 | 98.2 (58.9) | 97.0 (NA) | 5 | 78.8 (50.1) | 107.0 (NA) | 7 | 78.4 (45.7) | 94.5 (26.2) |
| BUN/Creatinine Ratio, M (SD) | 27 | 22.9 (11.0) | 20.7 (4.7) | 23 | 22.4 (6.9) | 19.2 (5.8) | 24 | 30.3 (24.6) | 26.1 (8.0) | 23 | 20.2 (7.9) | 18.4 (7.6) |
| Sodium (mmol/L), M (SD) | 38 | 139.4 (3.6) | 137.9 (3.5) | 31 | 140.6 (3.3) | 140.0 (2.8) | 25 | 132.3 (29.6) | 139.3 (3.2) | 24 | 139.2 (4.4) | 141.2 (1.9) |
| Potassium (mmol/L), M (SD) | 38 | 4.1 (0.5) | 4.1 (0.6) | 30 | 3.9 (0.6) | 4.0 (0.3) | 25 | 4.3 (1.3) | 4.5 (0.5) | 24 | 4.5 (1.0) | 4.2 (0.5) |
| Chloride (mmol/L), M (SD) | 38 | 103.3 (3.7) | 101.0 (4.4) | 31 | 103.3 (4.2) | 101.8 (4.1) | 25 | 102.5 (5.5) | 102.1 (2.1) | 24 | 101.9 (4.6) | 104.1 (1.4) |
| Carbon Dioxide, Total (mmol/L), M (SD) | 38 | 24.1 (4.8) | 25.3 (4.4) | 31 | 24.6 (4.0) | 25.3 (2.8) | 25 | 23.8 (3.5) | 26.3 (2.8) | 24 | 21.9 (3.8) | 25.4 (3.1) |
| Calcium (mg/dL), M (SD) | 38 | 8.4 (0.5) | 8.3 (0.7) | 31 | 8.3 (0.7) | 8.3 (0.8) | 25 | 8.4 (1.1) | 8.7 (0.9) | 24 | 8.6 (1.1) | 9.1 (0.4) |
| Protein, Total (g/dL), M (SD) | 33 | 5.9 (0.7) | 6.0 (0.8) | 30 | 5.7 (0.8) | 6.0 (0.7) | 25 | 5.6 (1.1) | 6.1 (0.6) | 22 | 6.0 (1.1) | 6.3 (0.4) |
| Albumin (g/dL), M (SD) | 35 | 3.4 (0.5) | 3.5 (0.6) | 30 | 3.2 (0.7) | 3.4 (0.6) | 25 | 3.2 (0.8) | 3.5 (0.8) | 22 | 3.4 (0.8) | 3.9 (0.4) |
| Globulin, Total (g/dL), M (SD) | 35 | 2.6 (0.5) | 2.6 (0.4) | 30 | 2.5 (0.5) | 2.6 (0.5) | 24 | 2.5 (0.5) | 2.6 (0.4) | 21 | 2.6 (0.6) | 2.3 (0.4) |
| A/G Ratio, M (SD) | 14 | 1.4 (0.3) | 1.3 (0.3) | 16 | 1.5 (0.4) | 1.5 (0.4) | 21 | 1.4 (0.3) | 1.4 (0.4) | 21 | 1.3 (0.4) | 1.7 (0.4) |
| Bilirubin, Total (mg/dL), M (SD) | 34 | 0.4 (0.2) | 0.4 (0.3) | 31 | 0.7 (0.8) | 0.4 (0.2) | 25 | 0.5 (0.2) | 0.5 (0.4) | 22 | 0.5 (0.2) | 0.6 (0.5) |
| Alkaline Phosphatase (IU/L), M (SD) | 35 | 84.9 (35.3) | 66.5 (21.6) | 31 | 101.8 (71.1) | 67.7 (22.1) | 25 | 83.9 (35.0) | 86.6 (15.2) | 22 | 94.2 (47.3) | 79.1 (19.3) |
| Aspartate aminotransferase (SGOT) (IU/L), M (SD) | 35 | 30.0 (18.3) | 38.2 (26.4) | 31 | 69.7 (117.3) | 47.3 (33.9) | 25 | 41.4 (52.7) | 46.1 (32.8) | 22 | 36.6 (20.0) | 27.6 (10.1) |
| Alanine aminotransferase (SGPT) (IU/L), M (SD) | 35 | 38.5 (39.7) | 57.2 (38.6) | 31 | 68.0 (78.8) | 107.0 (122.3) | 25 | 46.4 (39.7) | 108.7 (106.2) | 22 | 42.2 (29.3) | 70.8 (83.4) |

SD values “NA” denotes presence of only N=1 patient in that group/timepoint.

Supplementary Table 6. MSC quality control metrics for all four infusions (N=31 subjects, who received at least one infusion of 100MM HB-adMSCs).

| **Subject #** | **Infusion #** | **Product Lot** | **Total cell count (million)** | **Cell Viability (%)** | **CD73 (%)** | **CD29 (%)** | **CD31 (%)** | **CD45 (%)** |
| --- | --- | --- | --- | --- | --- | --- | --- | --- |
| HB-adMSC#1 | 1 | HB200020-01, P4ALR4 | 101 | 98.88 | 76.26 | 99.07 | 0.7 | 0.1 |
|  | 2 | HB200020-01, P4ALR5 | 120 | 95 | 77.53 | 99.97 | 0 | 0 |
|  | 3 | HB200020-01, P4ALR6 | 120 | 96.15 | 80.77 | 99.96 | 0 | 0.09 |
|  | 4 | HB200020-01, P4ALR7 | 120 | 93.92 | 95.5 | 100 | 0.05 | 0.05 |
| HB-adMSC#2 | 1 | HB200020-01, P4ALR4 | 101 | 98.88 | 76.26 | 99.07 | 0.7 | 0.1 |
|  | 2 | HB200020-01, P4ALR5 | 120 | 95 | 77.53 | 99.97 | 0 | 0 |
|  | 3 | HB200020-01, P4ALR6 | 120 | 96.15 | 80.77 | 99.96 | 0 | 0.09 |
|  | 4 | HB200020-01, P4ALR7 | 120 | 93.92 | 95.5 | 100 | 0.05 | 0.05 |
| HB-adMSC#3 | 1 | HB200020-01, P4ALR4 | 101 | 98.88 | 76.26 | 99.07 | 0.7 | 0.1 |
|  | 2 | HB200020-01, P4ALR5 | 120 | 95 | 77.53 | 99.97 | 0 | 0 |
|  | 3 | HB200020-01, P4ALR6 | 120 | 96.15 | 80.77 | 99.96 | 0 | 0.09 |
|  | 4 | HB200020-01, P4ALR7 | 120 | 93.92 | 95.5 | 100 | 0.05 | 0.05 |
| HB-adMSC#4 | 1 | HB200021-01, P4ALR1 | 93.3 | 90.52 | 98.995 | 99.96 | 0.04 | 0 |
|  | 2 | HB200021-01, P4ALR6 | 108 | 98.01 | 98.83 | 99.94 | 0.03 | 0.03 |
|  | 3 | HB200021-01, P4ALR7 | 92.4 | 96.95 | 95.85 | 99.85 | 0 | 0 |
|  | 4 | N/A |  |  |  |  |  |  |
| HB-adMSC#5 | 1 | HB200021-01, P4ALR1 | 93.3 | 90.52 | 98.995 | 99.96 | 0.04 | 0 |
|  | 2 | HB200021-01, P4ALR6 | 108 | 98.01 | 98.83 | 99.94 | 0.03 | 0.03 |
|  | 3 | HB200021-01, P4ALR8 | 108 | 96.03 | 93.07 | 99.95 | 0 | 0 |
|  | 4 | HB200021-01, P4ALR10 | 120 | 96.35 | 77.63 | 99.23 | 0 | 0.28 |
| HB-adMSC#6 | 1 | HB200021-01, P4ALR3 | 102 | 95.7 | 98.48 | 99.93 | 0 | 0 |
|  | 2 | N/A |  |  |  |  |  |  |
|  | 3 | N/A |  |  |  |  |  |  |
|  | 4 | N/A |  |  |  |  |  |  |
| HB-adMSC#7 | 1 | HB200021-01, P4ALR1 | 93.3 | 90.52 | 98.995 | 99.96 | 0.04 | 0 |
|  | 2 | HB200021-01, P4ALR5 | 119 | 95.1 | 97.4 | 99.92 | 0 | 0.04 |
|  | 3 | HB200021-01, P4ALR9 | 120 | 92.55 | 90 | 99.89 | 0 | 0 |
|  | 4 | HB200021-01, P4ALR10 | 120 | 96.35 | 77.63 | 99.23 | 0 | 0.28 |
| HB-adMSC#8 | 1 | HB200021-01, P4ALR2 | 117 | 96.41 | 99.81 | 99.95 | 0 | 0 |
|  | 2 | HB200021-01, P4ALR6 | 108 | 98.01 | 98.83 | 99.94 | 0.03 | 0.03 |
|  | 3 | HB200021-01, P4ALR9 | 120 | 92.55 | 90 | 99.89 | 0 | 0 |
|  | 4 | HB200021-01, P4ALR10 | 120 | 96.35 | 77.63 | 99.23 | 0 | 0.28 |
| HB-adMSC#9 | 1 | HB200021-01, P4ALR2 | 117 | 96.41 | 99.81 | 99.95 | 0 | 0 |
|  | 2 | HB200021-01, P4ALR5 | 119 | 95.1 | 97.4 | 99.92 | 0 | 0.04 |
|  | 3 | HB200021-01, P4ALR7 | 92.4 | 96.95 | 95.85 | 99.85 | 0 | 0 |
|  | 4 | HB200021-01, P4ALR12 | 100 | 95.83 | 79.99 | 99.9 | 0 | 0.04 |
| HB-adMSC#10 | 1 | HB200021-01, P4ALR2 | 117 | 96.41 | 99.81 | 99.95 | 0 | 0 |
|  | 2 | N/A |  |  |  |  |  |  |
|  | 3 | N/A |  |  |  |  |  |  |
|  | 4 | N/A |  |  |  |  |  |  |
| HB-adMSC#11 | 1 | HB200021-01, P4ALR2 | 117 | 96.41 | 99.81 | 99.95 | 0 | 0 |
|  | 2 | HB200021-01, P4ALR5 | 119 | 95.1 | 97.4 | 99.92 | 0 | 0.04 |
|  | 3 | HB200021-01, P4ALR7 | 92.4 | 96.95 | 95.85 | 99.85 | 0 | 0 |
|  | 4 | HB200021-01, P4ALR12 | 100 | 95.83 | 79.99 | 99.9 | 0 | 0.04 |
| HB-adMSC#12 | 1 | HB200021-01, P4ALR1 | 93.3 | 90.52 | 98.995 | 99.96 | 0.04 | 0 |
|  | 2 | HB200021-01, P4ALR5 | 119 | 95.1 | 97.4 | 99.92 | 0 | 0.04 |
|  | 3 | HB200021-01, P4ALR9 | 120 | 92.55 | 90 | 99.89 | 0 | 0 |
|  | 4 | HB200021-01, P4ALR10 | 120 | 96.35 | 77.63 | 99.23 | 0 | 0.28 |
| HB-adMSC#13 | 1 | HB200021-01, P4ALR2 | 117 | 96.41 | 99.81 | 99.85 | 0 | 0 |
|  | 2 | HB200021-01, P4ALR6 | 108 | 98.01 | 98.83 | 99.94 | 0.03 | 0.03 |
|  | 3 | HB200021-01, P4ALR9 | 120 | 92.55 | 90 | 99.89 | 0 | 0 |
|  | 4 | HB200021-01, P4ALR10 | 120 | 96.35 | 77.63 | 99.23 | 0 | 0.28 |
| HB-adMSC#14 | 1 | HB200021-01, P4ALR1 | 93.3 | 90.52 | 98.995 | 99.96 | 0.04 | 0 |
|  | 2 | HB200021-01, P4ALR4 | 107 | 98.04 | 96.93 | 100 | 0 | 0.06 |
|  | 3 | N/A |  |  |  |  |  |  |
|  | 4 | N/A |  |  |  |  |  |  |
| HB-adMSC#15 | 1 | HB200021-01, P4ALR1 | 93.3 | 90.52 | 98.995 | 99.96 | 0.04 | 0 |
|  | 2 | HB200021-01, P4ALR4 | 107 | 98.04 | 96.93 | 100 | 0 | 0.06 |
|  | 3 | HB200021-01, P4ALR7 | 92.4 | 96.95 | 95.85 | 99.85 | 0 | 0 |
|  | 4 | N/A |  |  |  |  |  |  |
| HB-adMSC#16 | 1 | HB200018-01, P4ALR10 | 115 | 96.85 | 83.42 | 99.84 | 0 | 0.04 |
|  | 2 | HB200018-01, P4ALR11 | 120 | 96.05 | 85.64 | 99.58 | 0.15 | 0.04 |
|  | 3 | N/A |  |  |  |  |  |  |
|  | 4 | N/A |  |  |  |  |  |  |
| HB-adMSC#17 | 1 | HB200016-01, P4ALR6 | 111 | 92.35 | 93.27 | 100 | 0 | 0.04 |
|  | 2 | HB200016-01, P4ALR10 | 118 | 97.59 | 92.14 | 99.94 | 0.02 | 0.06 |
|  | 3 | HB200016-01, P4ALR8 | 115 | 97.29 | 83.61 | 99.84 | 0 | 0 |
|  | 4 | HB200016-01, P4ALR11 | 109 | 97.79 | 91.94 | 99.93 | 0.07 | 0.1 |
| HB-adMSC#18 | 1 | HB200018-01, P4ALR10 | 115 | 96.85 | 83.42 | 99.84 | 0 | 0.04 |
|  | 2 | HB200018-01, P4ALR12 | 117 | 96.88 | 94.57 | 99.97 | 0 | 0.03 |
|  | 3 | HB200018-01, P4ALR14 | 120 | 96.98 | 92.68 | 99.97 | 0.03 | 0 |
|  | 4 | HB200018-01, P4ALR15 | 120 | 93.77 | 80 | 99.92 | 0.08 | 0 |
| HB-adMSC#19 | 1 | HB200018-01, P4ALR9 | 111 | 96.23 | 77.35 | 99.89 | 0.03 | 0.03 |
|  | 2 | HB200018-01, P4ALR9 | 111 | 96.23 | 77.35 | 99.89 | 0.03 | 0.03 |
|  | 3 | HB200018-01, P4ALR11 | 120 | 96.05 | 85.64 | 99.58 | 0.15 | 0.04 |
|  | 4 | HB200018-01, P4ALR15 | 120 | 93.77 | 80 | 99.92 | 0.08 | 0 |
| HB-adMSC#20 | 1 | HB200018-01, P4ALR10 | 115 | 96.85 | 83.42 | 99.84 | 0 | 0.04 |
|  | 2 | HB200018-01, P4ALR9 | 111 | 96.23 | 77.35 | 99.89 | 0.03 | 0.03 |
|  | 3 | HB200018-01, P4ALR13 | 118 | 96.9 | 92.79 | 100 | 0.03 | 0 |
|  | 4 | HB200018-01, P4ALR15 | 120 | 93.77 | 80 | 99.92 | 0.08 | 0 |
| HB-adMSC#21 | 1 | HB200016-01, P4ALR6 | 111 | 92.35 | 93.27 | 100 | 0 | 0.04 |
|  | 2 | HB200016-01, P4ALR10 | 118 | 97.59 | 92.14 | 99.94 | 0.02 | 0.06 |
|  | 3 | HB200016-01, P4ALR8 | 115 | 97.29 | 83.61 | 99.84 | 0 | 0 |
|  | 4 | HB200016-01, P4ALR12 | 105 | 96.72 | 86.95 | 99.97 | 0.06 | 0.03 |
| HB-adMSC#22 | 1 | HB200016-01, P4ALR6 | 111 | 92.35 | 93.27 | 100 | 0 | 0.04 |
|  | 2 | HB200016-01, P4ALR10 | 118 | 97.59 | 92.14 | 99.94 | 0.02 | 0.06 |
|  | 3 | HB200016-01, P4ALR8 | 115 | 97.29 | 83.61 | 99.84 | 0 | 0 |
|  | 4 | HB200016-01, P4ALR12 | 105 | 96.72 | 86.95 | 99.97 | 0.06 | 0.03 |
| HB-adMSC#23 | 1 | HB200016-01, P4ALR6 | 111 | 92.35 | 93.27 | 100 | 0 | 0.04 |
|  | 2 | HB200016-01, P4ALR10 | 118 | 97.59 | 92.14 | 99.94 | 0.02 | 0.06 |
|  | 3 | HB200016-01, P4ALR8 | 115 | 97.29 | 83.61 | 99.84 | 0 | 0 |
|  | 4 | HB200016-01, P4ALR12 | 105 | 96.72 | 86.95 | 99.97 | 0.06 | 0.03 |
| HB-adMSC#24 | 1 | HB200016-01, P4ALR6 | 111 | 92.35 | 93.27 | 100 | 0 | 0.04 |
|  | 2 | HB200016-01, P4ALR10 | 118 | 97.59 | 92.14 | 99.94 | 0.02 | 0.06 |
|  | 3 | HB200016-01, P4ALR8 | 115 | 97.29 | 83.61 | 99.84 | 0 | 0 |
|  | 4 | HB200016-01, P4ALR12 | 105 | 96.72 | 86.95 | 99.97 | 0.06 | 0.03 |
| HB-adMSC#25 | 1 | HB200020-01, P4ALR11 | 116 | 91.71 | 76.74 | 100 | 0.03 | 0 |
|  | 2 | N/A |  |  |  |  |  |  |
|  | 3 | N/A |  |  |  |  |  |  |
|  | 4 | N/A |  |  |  |  |  |  |
| HB-adMSC#26 | 1 | HB200020-01, P4ALR11 | 116 | 91.71 | 76.74 | 100 | 0.03 | 0 |
|  | 2 | N/A |  |  |  |  |  |  |
|  | 3 | N/A |  |  |  |  |  |  |
|  | 4 | N/A |  |  |  |  |  |  |
| HB-adMSC#27 | 1 | HB200020-01, P4ALR11 | 116 | 91.71 | 76.74 | 100 | 0.03 | 0 |
|  | 2 | N/A |  |  |  |  |  |  |
|  | 3 | N/A |  |  |  |  |  |  |
|  | 4 | N/A |  |  |  |  |  |  |
| HB-adMSC#28 | 1 | HB200020-01, P4ALR11 | 116 | 91.71 | 76.74 | 100 | 0.03 | 0 |
|  | 2 | N/A |  |  |  |  |  |  |
|  | 3 | N/A |  |  |  |  |  |  |
|  | 4 | N/A |  |  |  |  |  |  |
| HB-adMSC#29 | 1 | HB200020-01, P4ALR11 | 116 | 91.71 | 76.74 | 100 | 0.03 | 0 |
|  | 2 | N/A |  |  |  |  |  |  |
|  | 3 | N/A |  |  |  |  |  |  |
|  | 4 | N/A |  |  |  |  |  |  |
| HB-adMSC#30 | 1 | HB200020-01, P4ALR11 | 116 | 91.71 | 76.74 | 100 | 0.03 | 0 |
|  | 2 | N/A |  |  |  |  |  |  |
|  | 3 | N/A |  |  |  |  |  |  |
|  | 4 | N/A |  |  |  |  |  |  |
| HB-adMSC#31 | 1 | HB200020-01, P4ALR10 | 120 | 96.74 | 81.4 | 99.97 | 0 | 0.03 |
|  | 2 | N/A |  |  |  |  |  |  |
|  | 3 | N/A |  |  |  |  |  |  |
|  | 4 | N/A |  |  |  |  |  |  |

# Allogeneic HB-adMSCs were manufactured using four eligible donors’ cells and each product lot was manufactured for multiple patient administrations within its expiry. All product lots met the internal quality standards of Hope Biosciences, LLC prior to release. The approved dose of 1 x 10^8^ (± 20%) live cells (cell viability higher than or equal to 90.52%) were suspended in 10mL saline for each patient administration. MSCs are expected to be positive for CD73 and CD29 and negative for CD45 and CD31 surface markers.

**Supplementary Table 7.** Sample Randomization Spreadsheet.

| **SYMPTOM LEVEL** | **PRE-EXISTING CONDITION** | **ETHNICITY** | **AGE GROUP** | **TREATMENT GROUP** | **ORDER** |
| --- | --- | --- | --- | --- | --- |
| Mild | Yes | African American or Hispanic | Less than 41 | Treatment A | 1 |
| Mild | Yes | All Other | Less than 41 | Treatment A | 2 |
| Mild | No | African American or Hispanic | Less than 41 | Treatment A | 3 |
| Mild | No | All Other | Less than 41 | Treatment A | 4 |

Group assignment was determined by the Randomization spreadsheet, based upon the four stratification factors, using the formula:

=IF(COUNTA(K3:N3)=4,

CHOOSE(

MOD(

COUNTIFS($K$2:K3,K3,$L$2:L3,L3,$M$2:M3,M3,$N$2:N3,N3),2)+1,

"Treatment A","Treatment B")

,"")

# References used in Supplementary Material

1. Goodman SN. Introduction to Bayesian methods I: measuring the strength of evidence. Clin Trials. 2005 Aug 3;2(4):282–90.

2. Berry DA. Bayesian clinical trials. Nat Rev Drug Discov. 2006 Jan;5(1):27–36.

3. R Core Team. R: A Language and Environment for Statistical Computing. 2021.

4. Bürkner PC. brms: An R package for Bayesian multilevel models using Stan. J Stat Softw. 2017;80(1):1–28.

5. Stan Development Team. RStan: the R interface to Stan. 2020.

6. Best NG, Thomas A. Bayesian Graphical Models and Software for GLMs. In Marcel Dekker; 2000.

7. Gelman A. Prior distributions for variance parameters in hierarchical models (comment on article by Browne and Draper). Bayesian Anal [Internet]. 2006 Sep 1;1(3):515–34. Available from: https://projecteuclid.org/journals/bayesian-analysis/volume-1/issue-3/Prior-distributions-for-variance-parameters-in-hierarchical-models-comment-on/10.1214/06-BA117A.full
